# A visible-light photoactivatable di-nuclear Pt^IV^ triazolato azido complex†
†Electronic supplementary information (ESI) available: Synthetic details and characterisation data including X-ray crystallographic tables. CCDC 1885195 contains the supplementary crystallographic data for this paper. For ESI and crystallographic data in CIF or other electronic format see DOI: 10.1039/c9cc05310g


**DOI:** 10.1039/c9cc05310g

**Published:** 2019-09-02

**Authors:** Kezi Yao, Arnau Bertran, Alison Howarth, Jose M. Goicoechea, Samuel M. Hare, Nicholas H. Rees, Mohammadali Foroozandeh, Alice M. Bowen, Nicola J. Farrer

**Affiliations:** a Chemistry Research Laboratory , University of Oxford , 12 Mansfield Road , Oxford , OX1 3TA , UK . Email: Nicola.Farrer@chem.ox.ac.uk ; Tel: +44 (0)1865 285131

## Abstract

A novel Pt^IV^ triazolato azido complex **[3]-[N1,N3]** has been synthesised *via* a strain-promoted double-click reaction (SPDC) between a Pt^IV^ azido complex (**1**) and the Sondheimer diyne (**2**). Photoactivation of **[3]-[N1,N3]** with visible light (452 nm) in the presence of 5′-guanosine monophosphate (5′-GMP) produced both Pt^IV^ and Pt^II^ 5′-GMP species; EPR spectroscopy confirmed the production of both azidyl and hydroxyl radicals. Spin-trapping of photogenerated radicals – particularly hydroxyl radicals – was significantly reduced in the presence of 5′-GMP.

## Abstract 2

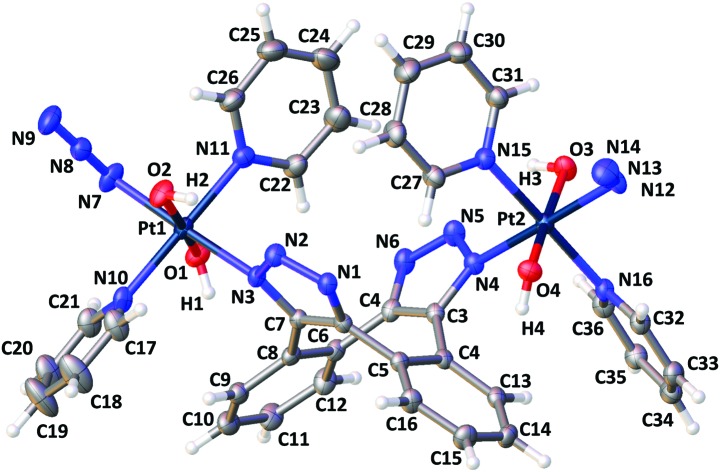
A novel Pt^IV^ triazolato azido complex **[3]-[N1,N3]** has been synthesised *via* a strain-promoted double-click reaction (SPDC) between a Pt^IV^ azido complex (**1**) and the Sondheimer diyne (**2**).

## 

Of the cancer patients who are treated with chemotherapy, approximately 50% receive a Pt^II^ drug such as cisplatin, carboplatin or oxaliplatin.^1^ However, the side-effects of treatment with Pt^II^ drugs can be severe, and the development of resistance can also be a serious problem.^2^ Octahedral low-spin 5d^6^ Pt^IV^ prodrugs are more kinetically inert than their Pt^II^ counterparts, and have the potential to address these issues.^3^–^6^ Both redox-activatable^7^,^8^ and photo-activatable^9^–^12^ Pt^IV^ prodrugs can exhibit promising pharmacological properties and can also incorporate ligands which - when released upon reduction of Pt^IV^ to Pt^II^ – exert an anti-cancer effect through mechanisms of action which are different from those of established Pt^II^-based drugs.^5^,^13^

Whilst the photochemistry of Pt^IV^ diazido complexes has been extensively investigated, Pt^IV^ monoazido complexes are less well explored. It was not known if two azido groups are necessary for photoreduction to Pt^II^, and what products were likely to be formed under irradiation. Direct derivation of a Pt^IV^ diazido complex was anticipated to be an effective way to answer these questions. Cycloaddition (click) reactions of metal azido complexes are well-established^14^ – although mostly for Pt^II^ rather than Pt^IV^. We recently reported the first Pt^IV^ triazolato monoazido complexes; synthesised *via* click reactions of Pt^IV^ azido complexes with both electron-deficient (*e.g.* 1,4-diphenyl-2-butyne-1,4-dione)^15^ and strained alkynes (*e.g.* DBCO; dibenzocyclooctyne-amine).^16^ Due to the popularity of click chemistry, a range of 1,2,3-triazoles with potential biomedical applications have been reported;^17^–^19^ 1,2,3-triazoles have the potential to participate in C–H hydrogen bonding; behave as hydrogen bond donors through both non-coordinated N-atoms; act as intercalating agents *via* π–π stacking and substitute for amides; making them attractive ligands.^20^ Pt^II^ triazole complexes^21^ and triazolato-bridged Pt^II^ complexes have been shown to demonstrate promising anti-cancer activity.^22^

Strain-promoted azide–alkyne [3+2] cycloaddition (SPAAC) exploits the spontaneous reactivity of cyclooctynes and azides due to inherent ring strain in the cyclooctyne.^23^ It can be used to assemble constructs under mild conditions for both biological (*e.g.* vascularly-targeted radiolabelled liposomes,^24^ glycan imaging^25^ and glycocalyx selective editing^26^) and chemical (*e.g.* Ru azido DBCO^27^ and Pt^II^-DBCO fluorophore^28^) applications.

The Sondheimer diyne (5,6,11,12-tetradehydrodibenzo[*a*,*e*]cyclooctene) (**2**) (Scheme 1) is a strained diyne which is straightforward to synthesise. It has been used as a monomer in Mo-catalysed ring-opening alkyne metathesis polymerization reactions^29^ and to couple together Ag(i) species^30^ and biomolecules.^31^ For our purposes, it enables the union of two – potentially different – Pt^IV^ azido complexes under catalyst-free conditions, without interaction of either Pt^IV^ centre with any other functional groups on the newly formed 1,2,3-triazole ligands – something which has complicated our earlier studies.^15^,^16^ Di-nuclear Pt^IV^ complexes are promising since they can be used to deliver multiple different biologically active agents to cancer cells.^8^ Here we report the facile, catalyst-free assembly of the water-soluble, water-stable, di-nuclear Pt^IV^ 1,2,3-triazolato azido complex **3-[N1,N3]** and our investigations into its photochemical properties (Scheme 1).

**Scheme 1:**
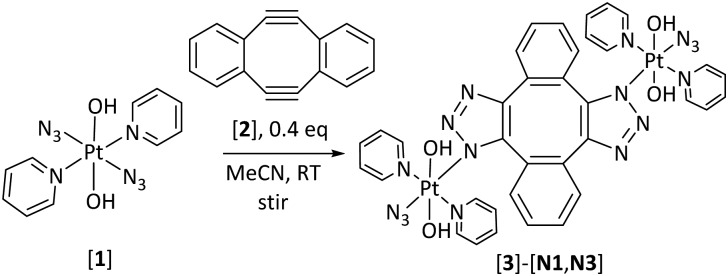
Synthesis of Pt^IV^ triazolato azido complex **(3)-[N1,N3]**.

Diyne (**2**) was synthesised according to literature reports and purified by column chromatography (ESI†).^32^,^33^ *trans*,*trans*,*trans*-[Pt(N_3_)_2_(OH)_2_(py)_2_] (**1**) was synthesised and purified by HPLC.^34^ The reaction between **1** (200 mg, 0.42 mmol) and **2** (30 mg, 0.15 mmol, 0.4 eq.) in MeCN (150 ml) at room temperature was monitored by LCMS and was judged to be complete after 2d. No mono-Pt^IV^ cycloaddition intermediates were detected by ESI-MS during the course of the reaction. This is consistent with DFT calculations of the reactivity profile of **2**, which indicate that the activation energy for the second cycloaddition is lower than for the first, due to the highly distorted alkyne bond in the mono-substituted intermediate, arising from steric repulsion between the substituent on the triazole ring and the hydrogen atom on the benzene ring.^31^ The reaction solution was dilute, minimising the formation of Pt^IV^ oligomers (see Fig. S1, ESI†) due to potential reactivity of the second Pt^IV^-azido ligand.

The major product (**3**) was detected by LCMS, as both [**3** + H]^+^ (1143.20 *m*/*z*) and [**3** + Na]^+^ (1165.36 *m*/*z*) adducts. Complex **3** was isolated by mass-directed LCMS as a mixture of two regioisomers: **3-[N1,N3]** and **3-[N3,N3]** (Fig. S2, ESI†). HPLC re-injection confirmed the isomers co-eluted with a purity of 95% (Fig. S3, ESI†). Following solvent removal and reconstitution of the pale yellow solid in *d*_3_-MeCN, ^1^H NMR spectroscopy indicated that **3-[N1,N3]** – which has two-fold symmetry – was the major isomer present. This is consistent with the previously reported reaction of **2** with excess benzyl azide which resulted in a 60% [N1,N3]: 38% [N1,N1] product distribution.^31^ Yellow crystals of **3-[N1,N3]** rapidly formed from the solution of regioisomers in *d*_3_-MeCN, on standing for 24 h.

Recrystallisation from MeCN afforded X-ray crystallographic quality crystals of **3-[N1,N3]** (Fig. 1), confirming [N1,N3] Pt^IV^-triazole coordination and revealing the ligand interactions around the puckered chair of the cyclooctene ligand. Distances between pyridine, triazole and benzene groups are shown in Fig. S4–S6 (ESI†); the pyridine ligands undergo π–π interactions with the cyclooctene ring ranging from 3.527–4.509 Å in length. A hydrogen-bond interaction of 2.178(3) Å was observed between Pt(1)–OH(2) and triazole N(2); the corresponding hydrogen-bond interaction on the other side of the molecule measured 2.242 Å (Pt–OH(3) to triazole N(5)). The identity of **3** was also confirmed by HRMS [**3** + H]^+^ (C_36_H_32_N_16_O_4_Pt_2_H): 1143.2123 *m*/*z* found; 1143.2069 *m*/*z* calcd (Fig. S7, ESI†).

**Fig. 1:**
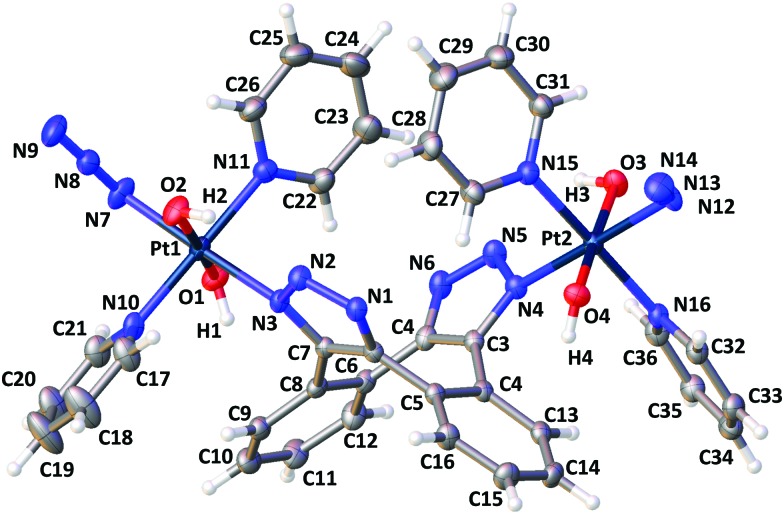
X-ray crystallographic structure of **3-[N1,N3]** with thermal ellipsoids displayed at 50% probability.^35^ Selected bond lengths (Å): Pt1–N3: 2.060(2), Pt1–N7: 2.041(3), N7–N8: 1.219(4), N8–N9: 1.151(5). Pt1–O2: 2.002(2). Selected angles (°): Pt1–N7–N8: 114.1(2); N7–N8–N9: 174.5(4). (See Tables S1–S3, ESI†).

Collision-induced dissociation (MS/MS) experiments of [**3-[N1,N3]** + H]^+^ demonstrated that at low collision energies the complex readily fragmented through loss of OH and N_3_ ligands, to give stable species [**3-[N1,N3]**–N_3_OH + H]^+^ (1083.32 *m*/*z*) and [**3-[N1,N3]**–2(N_3_OH)]^+^, (1023.30 *m*/*z*), consistent with our previous observations of azido ligand loss during MS/MS fragmentation of **1**.^34^ Stable mono-Pt fragments were also observed, including [Pt(C_16_N_6_H_7_)N_3_]^+^ (520.15 *m*/*z*) resulting from ejection of several small ligands and one Pt fragment from the central cyclooctene ligand, as well as smaller fragments including ([Pt(py)_2_(OH)_2_]^+^ (387.12 *m*/*z*)) ([Pt(py)_2_(OH)]^+^ (370.11 *m*/*z*)) and ([Pt(Py)_2_]^+^ (352.10 *m*/*z*)) demonstrating cleavage of the Pt–triazole bond (Fig. S7, ESI†).

Complex **3-[N1,N3]** was fully characterised by ^195^Pt, ^1^H and ^13^C NMR spectroscopic methods. ^1^H NMR spectroscopy revealed four different phenyl environments; a 2D ^1^H TOCSY experiment was used to determine the complete spin systems and to obtain coupling constants for overlapping signals (Fig. S9, ESI†) and 1D NOESY experiments revealed nOe interactions between pyridine (H_o_) and phenyl ring (H_A_) protons, confirming the regiochemistry of the product (Fig. S10, ESI†). ^13^C NMR spectral assignment (Fig. S11, ESI†) was aided by ^1^H–^13^C HSQC and HMBC experiments. Complex **3-[N1,N3]** gave rise to a single ^195^Pt NMR spectral resonance at 723 ppm (*d*_3_-MeCN, Fig. S12-top, ESI†).

Whilst **3-[N1,N3]** is stable in both *d*_3_-MeCN and D_2_O for a period of at least 5 weeks as judged by ^1^H NMR spectroscopy, the resonances change position markedly in the different solvents. Solvent removal from a sample of **3-[N1,N3]** in *d*_3_-MeCN followed by reconstitution in D_2_O resulted in an overall 163 ppm upfield shift in ^195^Pt NMR resonance from 723 ppm (*d*_3_-MeCN) to 857 ppm (1 : 1 *d*_3_-MeCN : D_2_O) to 886 ppm (D_2_O, Fig. S12-bottom, ESI†). In the ^1^H NMR spectrum (D_2_O), the H_A′_ and H_B′_ protons of the benzene rings no longer superimposed on the pyridyl H_m_ resonances (Fig. S13 and S14, ESI†). Consistent with this, the ^195^Pt NMR resonance of **1** also changes by 164 ppm on changing solvent from *d*_3_-MeCN (778 ppm, this work) to D_2_O (942 ppm).^34^ Selective ^1^H NOESY NMR experiments on **3-[N1,N3]** (D_2_O) revealed the same nOe correlations which were observed in *d*_3_-MeCN, with dissolution in 1 : 1 MeCN/D_2_O showing ^1^H NMR resonances at intermediate chemical shifts (Fig. S14-middle, ESI†), indicating that the change is unlikely to be due to a formal N1–N2 Pt–triazole rearrangement in D_2_O – a possibility for metal triazole complexes which we wanted to rule out.^27^,^36^,^37^

IR spectroscopy of a *d*_3_-MeCN sample of **3-[N1,N3]** (Fig. S15, ESI†) showed a strong *ν*_asym_N_3_ stretch at 2043 cm^–1^; slightly lower than observed for **1** (2051 cm^–1^, solid).^38^ The UV-Vis spectrum of **3-[N1,N3]** showed a long shoulder with *λ*_max_*ca.* 254 nm tailing into the visible region corresponding to the N_3_ → Pt LMCT transition band, with increased intensity at shorter wavelengths compared to **1** due to the additional aromatic groups (Fig. S16, ESI†).

A D_2_O (1 ml) solution of complex **(3)-[N1,N3]** (5.6 mg) and the DNA model 5′-GMP (2 eq. 4.8 mg) was irradiated (*λ*_irr_ 452 nm) with regular monitoring by LCMS and ^1^H NMR spectroscopy. Both Pt^IV^ and Pt^II^ photoproducts were detected by LCMS including non-5′-GMP bound species (where M = **3-[N1,N3]**): [M–N_3_]^+^ at 1100.12 *m*/*z*; [M–H_2_O_2_ + H]^+^ at 1109.12 *m*/*z*; [**3-[N1,N3]**–N_3_OH + H^+^]^+^ 1084.05 *m*/*z*. The cyclic-5′-GMP species [Pt^II^(OH)(py)_2_(N_5_C_10_O_7_H_12_P)]^+^ was observed at 715.14 *m*/*z*, although – unlike for similar investigations with complex **1** – no evidence of [Pt(OH)(py)_2_(5′-GMP)]^+^ was observed (predicted 733.1097 *m*/*z*). The LCMS *m*/*z* range is limited to 1250 *m*/*z* and a different ESI-MS instrument was therefore used to detect the presence of higher mass species, including the Pt^II^*bis*-GMP adduct: [M–2(H_2_O_2_)–2N_3_ + 2(5′-GMP) + Na]^+^ at 1739.85 *m*/*z*. (Fig. S17, ESI†).

The photochemistry was also monitored by ^1^H–^195^Pt HMBC and 1D ^195^Pt NMR spectroscopy (**(3)-[N1,N3]** 22 mM; 5′-GMP 46 mM, 1 : 1 D_2_O : *d*_3_-MeCN, *λ*_irr_ 452 nm, 180 min). During irradiation, the intensity of the ^195^Pt NMR spectroscopic resonance corresponding to **(3)-[N1,N3]** (854 ppm) decreased, with small amounts of new Pt^IV^ species (1267, 1350 ppm) and two more intense Pt^II^ signals appearing (–2224 ppm and –2369 ppm; Fig. S18 and S19, ESI†). These spectra were consistent with the formation of multiple Pt^II^ and Pt^IV^ photoproducts, as observed by LCMS (for discussion see end of ESI†).

Irradiation of a solution of **3-[N1,N3]** (1.15 mM) and 5,5-dimethyl-1-pyrroline *N*-oxide (DMPO, 20 mM) monitored by EPR spectroscopy in either water or cell-free lysate (KNS42) generated azidyl (N_3_˙) and hydroxyl (OH˙) radical species, trapped in a 85 : 15 and 90 : 10 molar ratio, respectively (Fig. S20(a), ESI† and Fig. 2(a)), with a maximum trapped radical concentration of 7 μM. The signals started to decay after ∼30 min irradiation (Fig. S23(a), ESI† and Fig. 2(b)). The inclusion of 5′-GMP in the solution of **3-[N1,N3]** in lysate had a significant effect; the maximum trapped radical concentration reduced to 3 μM with a 95 : 5 N_3_˙ : OH˙ molar ratio (Fig. S22(a), ESI†) and radical trapping slowed down (Fig. S25(a), ESI†). These experiments were repeated with complex **1** (Fig. S20(b), S21(b) and S22(b), ESI†) which released almost no hydroxyl radicals, reaching a much higher trapped radical maximum concentration of 32 μM in water and lysate. The rise and decay of the signal was faster (Fig. S23(b) and S24(b), ESI†) in comparison to **3-[N1,N3]**, consistent with **1** having a greater absorbance at the wavelength of irradiation. The effect of including 5′-GMP in the lysate solution of complex **1** was less pronounced (Fig. S22(b) and S25(b), ESI†) than for **3-[N1,N3]**, with only a slightly lower maximum trapped radical concentration (28 μM) and slower kinetics in the presence of 5′-GMP. Minimal radical release was observed in the absence of irradiation in aqueous solution, consistent with the observed stability of **3-[N1,N3]** and **1** (Fig. S26, ESI†). Irradiation of controls (DMPO, and 5′-GMP + DMPO) in lysate did not result in any trapped radicals (Fig. S27, ESI†).

**Fig. 2:**
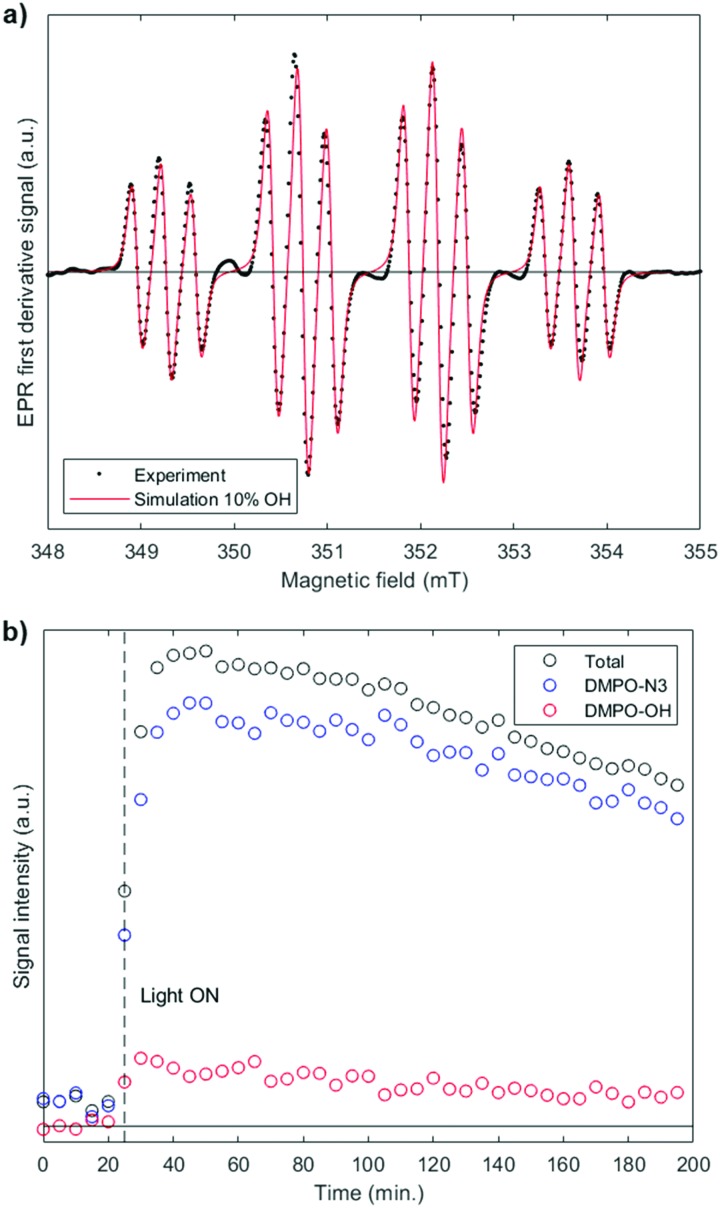
X-band cw-EPR spectrum (a) showing trapping of azidyl (N_3_˙) and hydroxyl (OH˙) radicals (1.15 mM **(3)-[N1,N3]** + 20 mM DMPO spin-trap) in freshly prepared KNS42 lysate (*λ*_irr_ 440–480 nm; spectra averaged over 1 h of maximum signal intensity); (b) fitted spectrum with 90% DMPO-N_3_ and 10% DMPO-OH (red line). The kinetic profile (b) for the total radical adduct signal (black) has been deconvoluted into the DMPO-N_3_ (blue) and DMPO-OH (red) contributions.

To conclude, we have demonstrated an effective method for joining together two Pt^IV^ azido complexes to give the di-nuclear Pt^IV^ triazolato azido complex **3-[N1,N3]** which is soluble and stable in aqueous solution for at least 5 weeks. Irradiation of **3-[N1,N3]** with visible light (*λ*_irr_ 452 nm) in the presence of 5′-GMP results in the formation of new Pt^IV^ and Pt^II^ species as well as radical species (N_3_˙, OH˙) in both H_2_O and cell-free lysate. Whilst the presence of two Pt-azido groups in complex **1** predominantly favours photochemical release of azido radicals, **3-[N1,N3]** undergoes photoreduction to Pt^II^ with the production of a greater proportion of hydroxyl radicals, consistent with the Pt^IV^ monoazido structure. Radical – particularly OH˙ – trapping from **3-[N1,N3]** was affected to a greater extent by the presence of 5′-GMP, in contrast to irradiation of **1**. It has previously been shown that N_3_˙ produced by irradiation of **1** can be quenched by l-tryptophan (Trp),^39^ forming Trp radicals;^40^ our future work will investigate the interaction of hydroxyl radicals with 5′-GMP and the possible photocytotoxicity of **3-[N1,N3]**.

We thank the Wellcome Trust (201406/Z/16/Z), Cancer Research UK (C5255/A18085) through the Cancer Research UK Oxford Centre, the John Fell Fund and L’Oréal (Women in Science Fellowship). NF thanks Prof. Stephen Faulkner and Prof. Andy Weller for helpful discussions and Dr Richard Hill and Prof. Chris Jones for the KNS42 cell line. EPR measurements were performed in the Centre for Advanced Electron Spin Resonance at the University of Oxford EPSRC (EPL011972/1). AMB thanks the Royal Society and EPSRC for a Dorothy Hodgkin Fellowship (DH160004); AMB and AB thank the Royal Society for a Grant for Research Fellows (RGF\R1\180099). MF thanks the Royal Society for a University Research Fellowship and an Enhancement Award (grant numbers URF\R1\180233 and RGF\EA\181018).

## Conflicts of interest

There are no conflicts to declare.

## Supplementary Material

Supplementary information

Crystal structure data
