# Supplementary material for: A visible-light photoactivatable di-nuclear PtIV triazolato azido complex
Source: Chem Commun (Camb). 2019 Sep 2;55(75):11287–90. doi: 10.1039/c9cc05310g (PMC6984334; doi:10.1039/c9cc05310g)
Supplement: Supplementary file 1 [file CC-055-C9CC05310G-s001.pdf]

Electronic Supplementary Material (ESI) for Chemical Communications.
This journal is © The Royal Society of Chemistry 2019

A visible-light photoactivatable di-nuclear Pt^{IV} triazolato azido complex

Kezi Yao, Arnau Bertran, Alison Howarth, Jose M. Goicoechea, Samuel M. Hare, Nicholas H. Rees, Mohammadali Foroozandeh, Alice Bowen, Nicola J. Farrer

Contents

Materials and Methods 1
Preparation of cell-free extract 3
Synthetic procedures 3
X ray crystallographic tables 7
HRMS 13
MS/MS 14
NMR spectroscopy 15
IR spectrum 19
UV-Vis Absorbance Spectroscopy 20
Photochemistry of 3-[N1,N3] 21
NMR Spectroscopy 22
EPR Spectroscopy 25

Materials and Methods

$K_2[PtCl_4]$ was purchased from Precious Metals Online. HPLC-grade solvents and Millipore-filtered H_2O were used for the preparation of compounds and purification by HPLC. All other chemical reagents were purchased from standard commercial vendors (*e.g.* Sigma-Aldrich or Alfa Aesar) and used as received. (IM) indicates use of a nylon syringe filter (pore size 0.2 µM). All manipulations were carried out under reduced lighting and solutions were prepared stored and handled with minimal exposure to light.

NMR spectroscopy. Due to the potential photosensitivity of the compounds, amberised NMR spectroscopy tubes (Goss Scientific/Norrell) were used. All spectra were acquired at 298 K unless otherwise stated, and processed using Topspin 3.2. All chemical shift (δ) values are given in parts per million and are referenced to residual solvent unless otherwise stated, *J* values are quoted in Hz. **1D ^{1}H NMR spectra:** were acquired on a Bruker AVIIIHD 500 MHz (500.13 MHz) equipped with a 5mm z-gradient broadband X-19F/1H BBFO SMART probe or a Bruker AVIIIHD 400 nanobay (400.17 MHz). **1D ^{13}C NMR and ^{195}Pt NMR spectra:** were acquired on a Bruker AVII 500 MHz spectrometer equipped with a z-gradient triple resonance inverse ^{1}H/^{19}F(^{13}C) TXI probe. ^{195}Pt chemical shifts were externally

referenced to K_2PtCl_6 in 1.5 mM HCl in D_2O (δ 0 ppm). **2D ultra-broadband ^{1}H-^{195}Pt HMBC NMR spectra**: were acquired on a Bruker AVIIIHD 500 MHz. The probe used was a 5mm broadband X-19F/1H BBFO SMART probe equipped with z-gradient, with 10.3 µs and 11.8 µs hard 90° pulses for ^{1}H and ^{195}Pt respectively. The ^{1}H-^{195}Pt HMBC experiment used a conventional phase cycled HMBC pulse sequence[1] in which two excitation pulses on ^{195}Pt were replaced by short ultra-broadband swept-frequency chirp pulses. The duration and bandwidth of the 90° chirp pulse used were set to 200 µs and 1 MHz respectively, with RF amplitude of 18.7 kHz, corresponding to an adiabaticity (Q) factor of 0.43. The spectral windows were set to 6.5 kHz (13 ppm) and 538 kHz (5006 ppm) for direct (^{1}H) and indirect (^{195}Pt) dimensions respectively. FIDs were acquired with 4096 complex points along the observe dimension (^{1}H) and 128 increments along the indirect dimension. Numbers of dummy scans (DS) and scans (NS) were both set to 16, with an overall recycle time (AQ+D1) of 414 ms, with a total experimental time of 12 min.

Mass Spectrometry: low resolution ESI-MS were obtained with a Waters Micromass LCT Premier XE spectrometer. **HRMS:** obtained with a Thermofisher Exactive Plus with a Waters Acuity UPLC system. **MS/MS experiments**: were performed on an Acuity UPLC in flow injection analysis mode, equipped with a Waters Xevo G25 QTOF. MS data were processed using MassLynx 4.0. **HPLC:** were performed with a Waters Autopurification system. **prep-HPLC**: used a Waters X-Bridge OBD semi-prep column (5 µm, 19 mm x 50 mm), with an injection loop of 1 ml, eluting with H_2O+0.1% NH_4OH (pH 9)/MeCN +0.1% NH_4OH. Samples (in H_2O/MeCN) were filtered (IM) and injected in 750 µL aliquots, with mass-directed purification with an ACQUITY QDa performance mass spectrometer. **Analytical HPLC:** used the same solvents and a Waters X-Bridge OBD column (5µm, 4.6 mm x 50 mm) and an injection loop of 0.02 ml. Retention times (t_R) are quoted for the solvent gradient: 0 min (95% A : 5% B); 1 min (95:5), 7.5 min (5:95) on the analytical column. **UV-visible absorption spectra:** were obtained with a T60U Spectrometer PG Instruments Ltd using UVWin Software, or the Waters HPLC. **Photochemistry**: samples were irradiated with stirring at a distance of 50 mm from a 5W MiniSun GU10 27 SMD LED bulb with an output centred at 452 nm (see **Figure S27**). **EPR spectroscopy:** solutions for EPR measurements were prepared at concentrations of 2.3 mM Pt and 20 mM DMPO in water, freshly prepared KNS42 (glioma) cell-free lysate or lysate containing 2.3 mM 5'-GMP. The solutions were loaded into quartz tubes (1.2 mm outer diameter) using metal needles and plastic syringes. The capillaries were placed in larger (4mm outer diameter) quartz tubes. Sample volume (7 µL) and position were adjusted so that the length of the sample matched the optical window of the resonator, for an optimal illumination. The EPR experimental setup consisted of a Xe arc lamp operated at 700 W, coupled to the resonator (X-band Super High Sensitivity Probehead, Bruker) of the EPR spectrometer (X-band EMXmicro, Bruker) through a liquid waveguide. The light was filtered before entering the waveguide, using a 410 nm long-pass filter followed by a 440 – 480 nm band pass filter. Measurements were carried out in continuous-wave mode, at X-band frequency (ca. 9.5 GHz) and room temperature, using the following parameters: field sweep of 8 mT, receiver gain of 30 dB, modulation amplitude of 0.1 mT and microwave attenuation of 23.0 dB. Quantification was done using the second integral of the spectra averaged for 1 h (**3**-[**N1**,**N3**]) and 10 min (**1**) under irradiation, by interpolation in a calibration curve of TEMPO solutions of known concentrations measured in the same conditions as the samples, in the dark. The spectra were fitted using a modified EasySpin[2] *garlic* function to allow two species to be fitted simultaneously. For the DMPO-N_3 adduct, isotropic hyperfine couplings to the nitroxidic nitrogen (a_{NO}^N = 1.450 mT), the β-proton ($a_β^H$ = 1.475 mT) and the α-nitrogen

of the trapped azidyl radical (a_{α}^{N} = 0.314 mT) were considered. For the DMPO-OH adduct, only the first two hyperfine couplings were included. An isotropic g-tensor (g =2 .00587 mT) was used in both cases.

Preparation of cell-free extract

The paediatric high grade glioma cell line KNS42 was grown in DMEM media (ThermoFisher, 61965-026) containing 10% foetal bovine serum (Sigma, F7524). KNS42 cells were grown in a CO_2 incubator (5% CO_2) at 37°C. To prepare the cell-free lysate, cell flasks were washed with 5mL HBSS (ThermoFisher, 14170120) and cells digested in 2mL of TryplExpress (ThermoFisher, 12604021). Following complete digestion, TryplEpress was inhibited with an equal volume of DMEM media and the cell suspension was centrifuged at 1200 rpm x 5 min at 4°C to form a pellet. The cell pellet was washed in dH_2O and centrifuged to pellet 3 times. The final cell pellet was resuspended in 1 ml of dH_2O (1.0 x 10^7 cells/ml). To ensure complete lysis, the cell suspension was minimally sonicated. Cell debris was removed by centrifugation at 13,0000 rpm for 15 min at 4°C. The cell-free lysate was kept on ice prior to running EPR experiments which were conducted as soon as possible after lysate preparation.

Synthetic procedures

***Caution!* No problems were encountered during this work, however heavy metal azides are known to be shock sensitive detonators, therefore it is essential that platinum azido complexes are handled with care. In addition, due to their photosensitivity, the platinum azido complexes (particularly solutions) were handled and stored with minimal light exposure.**

The Pt^{IV} complex *trans,trans,trans*–$[Pt(N_3)_2(OH)_2(py)_2]$ (**1**) was synthesised as previously reported and purified by HPLC before use.[3]

The Sonheimer diyne (5,6,11,12-Tetradehydrodibenzo[a,e]cyclooctene, **2**) was synthesised and purified by column chromatography as outlined below:[4,5]

***ortho*-(Phenylsulfonylmethyl)benzonitrile:** 3.92 g of *alpha*-bromotolunitrile (20 mmol) was added to benzene sulfinic acid sodium salt dihydrate (4.80 g, 24.0 mmol, *ChemCruz*) in DMF (30 ml) under an inert N_2 atmosphere. The reaction mixture was stirred at 80 ^{o}C for two hours and then cooled to room temperature. The reaction mixture was worked up with H_2O/EtOAc. The organic layer was isolated, dried with $MgSO_4$, filtered and the solvent removed *in vacuo* to yield 5.65 g of crude product. The crude product was then recrystallised with minimal hot EtOAc and hexane until the clouding point was reached, yielding 2.58 g of solid white product (10 mmol, 50%). ^{1}H NMR (400 MHz, $CDCl_3$) δ: 7.79 – 7.41 (m, 9H), 4.58 (s, 2H). ESI-MS (MeCN/H_2O) *m/z*: 280.03 $[M+Na]^+$.

***ortho*-(phenylsulfonylmethyl)benzaldehyde:** 2.58 g of *ortho*-(phenylsulfonylmethyl)benzonitrile (10 mmol) was dissolved in dry DCM and 32 ml (23 mmol) of diisobutylaluminium hydride (DIBALH) was added at −78 ^{o}C under an inert N_2 atmosphere. The reaction mixture was stirred at −78 ^{o}C for 2 hours, then 60 ml of 1 M aqueous NH_4Cl was poured onto it. 100 ml of 1 M HCl was added and the reaction solution washed with DCM (2 x 200 ml). The organic layers were combined, dried under $MgSO_4$ and filtered. The solvent was then removed *in vacuo*. The crude product was then filtered through a thin pad (silica gel and DCM), yielding 410 mg of pure product as a white solid (1.58 mmol, 16%). ^{1}H NMR (400 MHz, $CDCl_3$) δ: 9.81 (s, 1H). 7.78 - 7.42 (m, 10H), 5.02 (s, 2H). ESI-MS (MeOH) *m/z:* 260.05 $[M+H]^+$.

5,6,11,12-Tetradehydrodibenzo[a,e]cyclooctene (2): 410 mg (1.58 mmol) of *ortho*-(phenylsulfonylmethyl)benzaldehyde was added to $ClPO(OEt)_2$ (0.27 ml, 1.9 mmol) and dry THF (47

ml) under an inert N_2 atmosphere. Lithium bis(trimethylsilyl)amide (LiHMDS, 1 M in THF, 3.2 ml, 3.2 mmol) was added at -78 °C . The reaction mixture was stirred at -78 °C for 30 minutes, then at room temperature for 1.5 hours. Lithium diisopropylamide (LDA, 1 M, in THF/hexane, 7.9 ml, 7.9 mmol) was added dropwise at -78°C. The reaction mixture was stirred at -78°C for two hours and then quenched with aqueous NH_4Cl (100 ml, 1 M). The reaction mixture was worked up with H_2O (1 x 100 ml) and EtOAc (2 x 200 ml). The organic layers were combined and the solvent removed *in vacuo* to yield 870 mg of crude product. The product was purified by column chromatography (DCM:hexane 2:3) to yield 45 mg of pure product as a yellow solid (0.23 mmol, 11%). ^{1}H NMR (400 MHz, $CDCl_3$) δ: 6.92-6.95 (m, 4H), 6.71-6.77 (m, 4H). ESI-MS (MeCN /H_2O) *m/z*: 401.30 $[2M+H]^+$.

Synthesis of 3-[N1, N3]

Diyne **2** (5,6,11,12-Tetradehydrodibenzo[a,e]cyclooctene) (30 mg, 0.15 mmol, 0.4 eq) was added to *trans,trans,trans*-$[Pt(N_3)_2(OH)_2(py)_2]$ (200 mg, 0.42 mmol) in MeCN (150 ml) and the reaction mixture stirred at room temperature for 48 h. The resulting yellow solution was concentrated, filtered and purified by HPLC, the product **3** eluting as a single species at t_R = 3.7 min. The mixture of isomers of complex **3** was reconsituted in MeCN; crystals of **3 – [N1, N3]** formed on standing at room temperature (10 mg, 0.09 mmol, 12 %).

^{1}H NMR (500 MHz, d_3-MeCN) δ: 8.64 (dd, 8H, $^3J_{HPt}$ = 27.5, $^3J_{HH}$ = 6.2, H_o) 7.91 (dd, 4H, $^3J_{HH}$ = 7.3, $^3J_{HH}$ = 7.3, H_p), 7.45 (d, 2H, $^3J_{HH}$ = 5.8, $H_{A'}$), 7.41(dd, 8H, $^3J_{HH}$ = 6.7, $^3J_{HH}$ = 6.7, 8H_m), 7.39 (t, 2H, $^3J_{HH}$ = 8.3, $H_{B'}$), 7.03 (t, 2H, $^3J_{HH}$ = 7.5, 1H, H_B), 5.90 (d, 2H, $^3J_{HH}$ = 7.8, H_A).

^{13}C NMR (126 MHz, d_3-MeCN) δ: 150.1 (C_o), 147.3 (q, HMBC to $H_{A'}$, C_2), 142.9 (C_p), 141.0 (q, HMBC to H_A, C_3), 134.3 (q, HMBC to $H_{B'}$ and H_A, C_1), 131.5, (q, HMBC to $H_{A'}$, C_4), 130.8 ($C_{A'}$ HSQC to 7.44), 130.7 (HSQC to 5.90, C_A), 130.2 (HSQC to 7.39, $C_{B'}$), 128.9 (HSQC to 7.03, C_B), 126.9 (HSQC to H_m, $^4J_{13C-195Pt}$ = 27.3, C_m).

^{195}Pt NMR (107 MHz, d_3-MeCN) δ: 723.

^{195}Pt NMR (107 MHz, D_2O) δ: 886.

HRMS ($C_{36}H_{32}N_{16}O_4Pt_2H$) *m/z:* 1143.2123 found; 1143.2069 calcd. (4.7 ppm error).

MSMS (1143.21 $[(\mathbf{3})\text{-}[N1,N3]+H]^+$ = $[Pt_2(py)_4(OH)_4(C_{16}N_6H_8)(N_3)_2H]$)*m/z*: 1083.32, $[Pt_2(py)_4(OH)_3(C_{16}N_6H_8)(N_3)]^+$ = [(3)-[N1,N3] - $N_3OH]^+$), 1023.30 ($[Pt_2(py)_4(OH)_2(C_{16}N_6H_8)]$ = [(3)-[N1,N3] - 2.$N_3OH]^+$), 520.15 ($[Pt(C_{16}N_6H_7)N_3]^+$), 387.12 ($[Pt(py)_2(OH)_2]^+$, 370.11 ($[Pt(py)_2(OH)]^+$, 352.10 ($[Pt(py)_2]^+$).

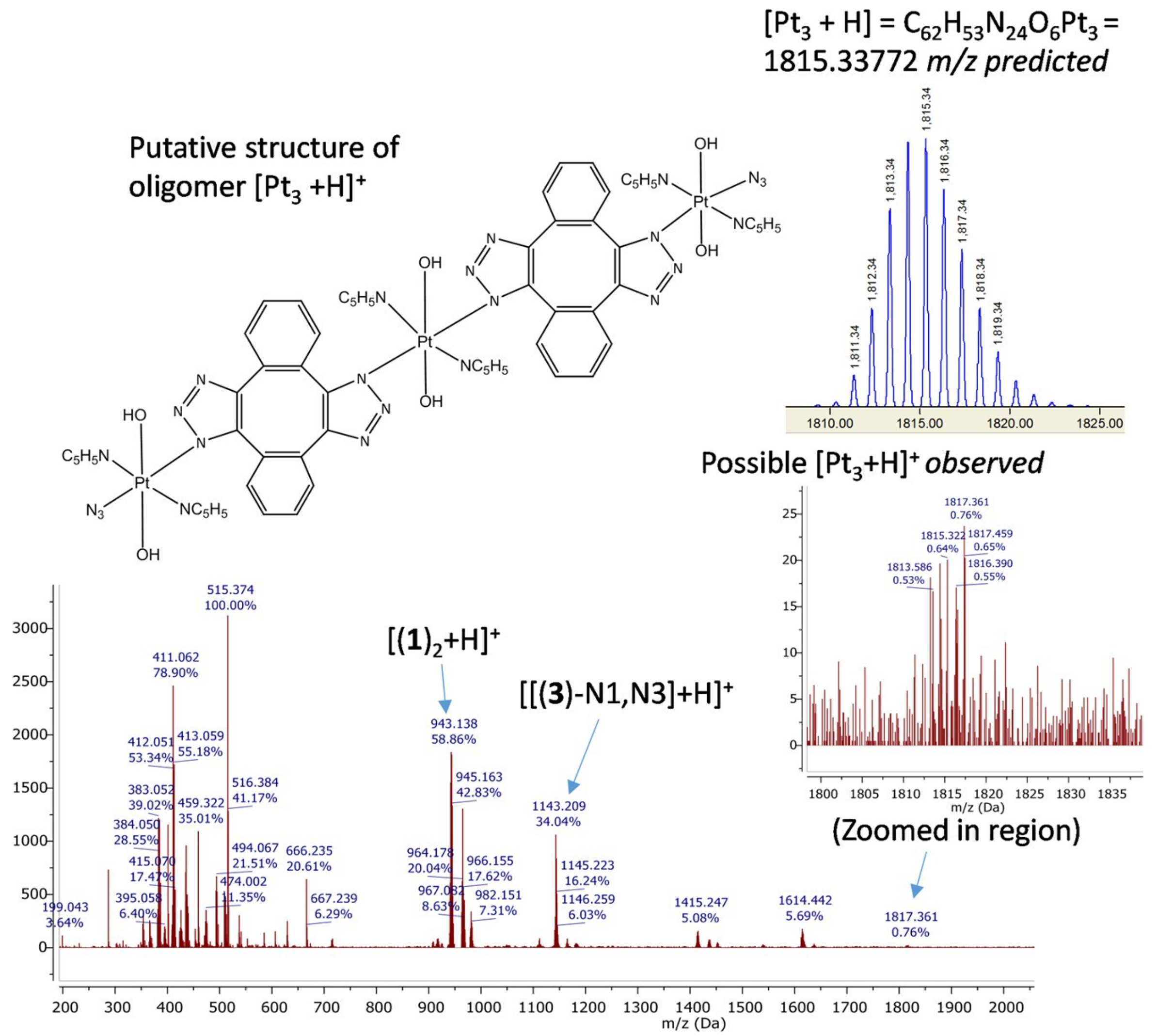

Figure S1. ESI-MS of reaction mixture between **1** and **2** before HPLC purification of (**3**)-[N1,N3]. Trace amounts of a possible trinuclear product ([Pt_3 + H] = $C_{62}H_{53}N_{24}O_6Pt_3$ = 1815.33772 *m/z* predicted) were detected by ESI-MS of the crude reaction mixture.

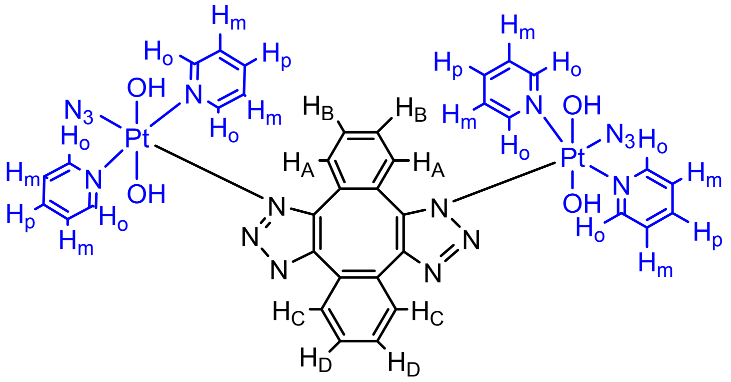

(3)-[N1, N1]

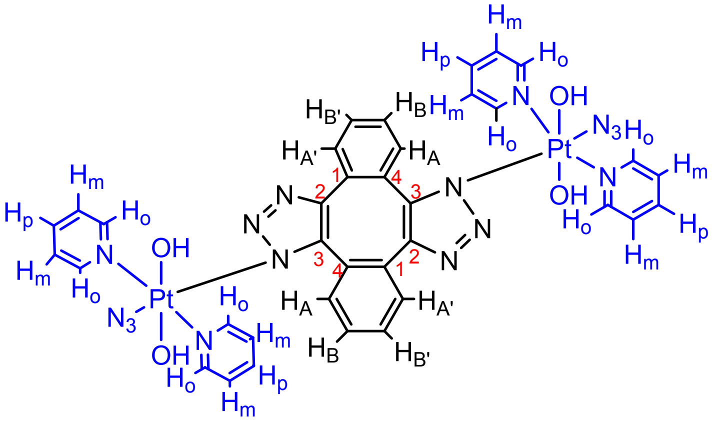

(3)-[N1, N3]

Figure S2. Structures of isomers (**3**)-[N1,N1] and (**3**)-[N1,N3]

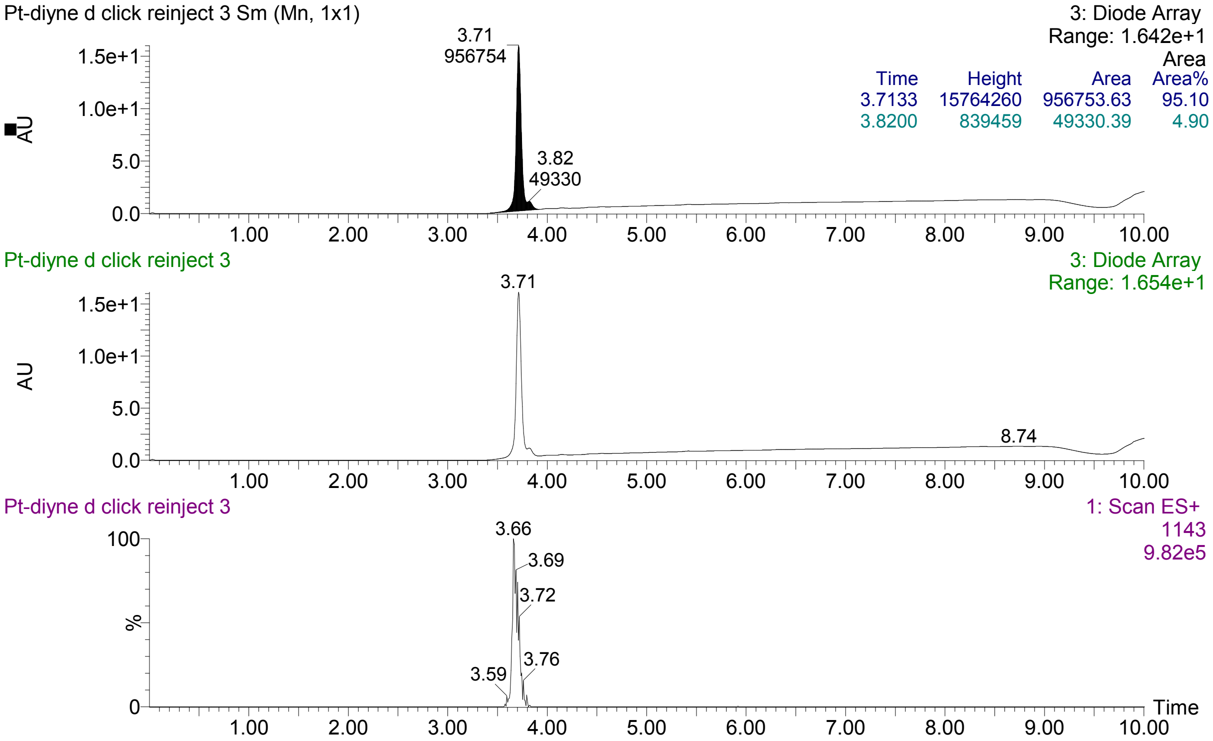

Figure S3. HPLC trace of reinjected complex **3-[N1,N3]** for purity testing. t_R = 3.7 min (**3-[N1,N3]**, 95%). Relative intensities are calculated by relative UV-Vis spectral absorbance integrated over the range 210 nm – 400 nm.

X ray crystallographic tables

Single crystals of (**3**)-[N1,N3] were grown from MeCN; the Pt complex co-crystallised with three molecules of MeCN in the unit cell.

Single crystal X-ray structure determination: Single-crystal X-ray diffraction data were collected using an Oxford Diffraction Supernova dual-source diffractometer equipped with a 135 mm Atlas CCD area detector. Crystals were selected under Paratone-N oil, mounted on micromount loops and quench-cooled using an Oxford Cryosystems open flow N2 cooling device. Data were collected at 150 K using mirror monochromated Cu Kα radiation (λ = 1.5418 Å; Oxford Diffraction Supernova) and processed using the CrysAlisPro package, including unit cell parameter refinement and inter-frame scaling (which was carried out using SCALE3 ABSPACK within CrysAlisPro).[6] Equivalent reflections were merged and diffraction patterns processed with the CrysAlisPro suite. Structures were subsequently solved using direct methods and refined on F2 using the SHELXL package.[7,8,9]

Table S1 Crystal data and structure refinement for (**3**)-[N1,N3]	
Identification code	CCDC 1885195
Empirical formula	$C_{42}H_{41}N_{19}O_4Pt_2$
Formula weight	1266.12
Temperature/K	150(2)
Crystal system	monoclinic
Space group	$P2_1/c$
a/Å	13.23900(10)
b/Å	16.72960(10)
c/Å	20.55360(10)
α/°	90
β/°	90.3440(10)
γ/°	90
Volume/Å^3	4552.20(5)
Z	4
ρ_{calc}g/cm^3	1.847
μ/mm^{-1}	11.872
F(000)	2456.0
Crystal size/mm^3	0.080 × 0.080 × 0.040
Radiation	CuKα (λ = 1.54184)
2Θ range for data collection/°	8.518 to 152.272
Index ranges	-16 ≤ h ≤ 16, -20 ≤ k ≤ 11, -25 ≤ l ≤ 25
Reflections collected	37582
Independent reflections	9459 [R_{int} = 0.0280, R_{sigma} = 0.0227]
Data/restraints/parameters	9459/9/622
Goodness-of-fit on F^2	1.130
Final R indexes [I>=2σ (I)]	R_1 = 0.0244, wR_2 = 0.0614
Final R indexes [all data]	R_1 = 0.0260, wR_2 = 0.0626
Largest diff. peak/hole / e Å^{-3}	2.08/-0.93

Table S2. Bond Lengths for (**3**)-[N1,N3]

Atom	Atom	Length/Å		Atom	Atom	Length/Å
Pt1	O2	2.002(2)		C4	C13	1.404(4)
Pt1	O1	2.022(2)		C4	C5	1.407(4)
Pt1	N11	2.026(3)		C5	C16	1.402(4)
Pt1	N10	2.029(3)		C5	C6	1.471(4)
Pt1	N7	2.041(3)		C6	C7	1.379(4)
Pt1	N3	2.060(2)		C7	C8	1.480(4)
Pt2	O4	2.005(2)		C8	C9	1.401(4)
Pt2	O3	2.006(2)		C9	C10	1.389(4)
Pt2	N15	2.025(3)		C10	C11	1.383(5)
Pt2	N16	2.034(2)		C11	C12	1.390(5)
Pt2	N12	2.041(2)		C13	C14	1.395(4)
Pt2	N4	2.052(2)		C14	C15	1.391(5)
N1	N2	1.321(4)		C15	C16	1.393(5)
N1	C6	1.352(4)		C17	C18	1.368(6)
N2	N3	1.344(3)		C18	C19	1.381(6)
N3	C7	1.353(4)		C19	C20	1.380(6)
N4	N5	1.347(4)		C20	C21	1.374(6)
N4	C3	1.368(4)		C22	C23	1.379(5)
N5	N6	1.314(4)		C23	C24	1.387(6)
N6	C2	1.359(4)		C24	C25	1.372(6)
N7	N8	1.219(4)		C25	C26	1.388(6)
N8	N9	1.151(5)		C27	C28	1.376(5)
N10	C17	1.347(4)		C28	C29	1.381(6)
N10	C21	1.350(4)		C29	C30	1.383(5)
N11	C26	1.346(4)		C30	C31	1.386(5)
N11	C22	1.350(4)		C32	C33	1.384(5)
N12	N13	1.220(4)		C33	C34	1.389(5)
N13	N14	1.144(4)		C34	C35	1.391(5)
N15	C31	1.342(4)		C35	C36	1.382(5)
N15	C27	1.355(4)				
N16	C36	1.347(4)				
N16	C32	1.349(4)				
C1	C12	1.398(4)				
C1	C8	1.407(4)				
C1	C2	1.472(4)				
C2	C3	1.378(4)				
C3	C4	1.479(4)				

Table S3. Bond Angles for **3**-[N1,N3]								
**Atom**	**Atom**	**Atom**	**Angle/˚**		**Atom**	**Atom**	**Atom**	**Angle/˚**
O2	Pt1	O1	172.47(9)		C12	C1	C8	119.1(3)
O2	Pt1	N11	90.97(11)		C12	C1	C2	120.7(3)
O1	Pt1	N11	87.89(10)		C8	C1	C2	120.2(3)
O2	Pt1	N10	89.84(11)		N6	C2	C3	108.9(3)
O1	Pt1	N10	91.24(10)		N6	C2	C1	123.0(3)
N11	Pt1	N10	179.00(10)		C3	C2	C1	128.0(3)
O2	Pt1	N7	88.09(11)		N4	C3	C2	104.8(3)
O1	Pt1	N7	84.49(11)		N4	C3	C4	124.5(2)
N11	Pt1	N7	91.03(12)		C2	C3	C4	130.7(3)
N10	Pt1	N7	88.41(12)		C13	C4	C5	119.6(3)
O2	Pt1	N3	89.86(9)		C13	C4	C3	120.7(3)
O1	Pt1	N3	97.52(9)		C5	C4	C3	119.6(3)
N11	Pt1	N3	87.71(10)		C16	C5	C4	119.2(3)
N10	Pt1	N3	92.88(10)		C16	C5	C6	120.4(3)
N7	Pt1	N3	177.58(11)		C4	C5	C6	120.4(3)
O4	Pt2	O3	173.16(9)		N1	C6	C7	109.2(2)
O4	Pt2	N15	86.83(10)		N1	C6	C5	122.1(2)
O3	Pt2	N15	90.72(10)		C7	C6	C5	128.7(3)
O4	Pt2	N16	92.31(9)		N3	C7	C6	104.9(2)
O3	Pt2	N16	90.15(10)		N3	C7	C8	124.6(2)
N15	Pt2	N16	179.13(11)		C6	C7	C8	130.4(3)
O4	Pt2	N12	86.06(10)		C9	C8	C1	119.5(3)
O3	Pt2	N12	87.55(10)		C9	C8	C7	120.0(3)
N15	Pt2	N12	90.00(11)		C1	C8	C7	120.4(3)
N16	Pt2	N12	90.14(10)		C10	C9	C8	120.2(3)
O4	Pt2	N4	96.62(9)		C11	C10	C9	120.4(3)
O3	Pt2	N4	89.77(9)		C10	C11	C12	119.9(3)
N15	Pt2	N4	90.43(10)		C11	C12	C1	120.8(3)
N16	Pt2	N4	89.48(10)		C14	C13	C4	120.4(3)
N12	Pt2	N4	177.30(10)		C15	C14	C13	119.9(3)
N2	N1	C6	107.5(2)		C14	C15	C16	120.1(3)
N1	N2	N3	109.0(2)		C15	C16	C5	120.6(3)
N2	N3	C7	109.4(2)		N10	C17	C18	121.0(3)
N2	N3	Pt1	114.87(18)		C17	C18	C19	119.5(4)
C7	N3	Pt1	134.72(19)		C20	C19	C18	119.5(4)
N5	N4	C3	109.1(2)		C21	C20	C19	118.7(4)
N5	N4	Pt2	117.45(19)		N10	C21	C20	121.5(3)
C3	N4	Pt2	132.9(2)		N11	C22	C23	121.6(3)
N6	N5	N4	109.2(2)		C22	C23	C24	118.8(4)
N5	N6	C2	107.9(2)		C25	C24	C23	119.5(4)
N8	N7	Pt1	114.1(2)		C24	C25	C26	119.4(3)
N9	N8	N7	174.5(4)		N11	C26	C25	121.1(3)
C17	N10	C21	119.7(3)		N15	C27	C28	121.1(3)
C17	N10	Pt1	120.3(2)		C27	C28	C29	119.5(3)
C21	N10	Pt1	120.0(2)		C28	C29	C30	119.1(3)
C26	N11	C22	119.5(3)		C29	C30	C31	119.3(3)
C26	N11	Pt1	120.4(2)		N15	C31	C30	121.1(3)
C22	N11	Pt1	120.1(2)		N16	C32	C33	120.7(3)
N13	N12	Pt2	113.8(2)		C32	C33	C34	119.5(3)
N14	N13	N12	174.8(3)		C33	C34	C35	119.0(3)
C31	N15	C27	119.8(3)		C36	C35	C34	119.1(3)
C31	N15	Pt2	120.3(2)		N16	C36	C35	121.2(3)
C27	N15	Pt2	119.9(2)					
C36	N16	C32	120.3(3)					
C36	N16	Pt2	119.7(2)					
C32	N16	Pt2	119.5(2)					

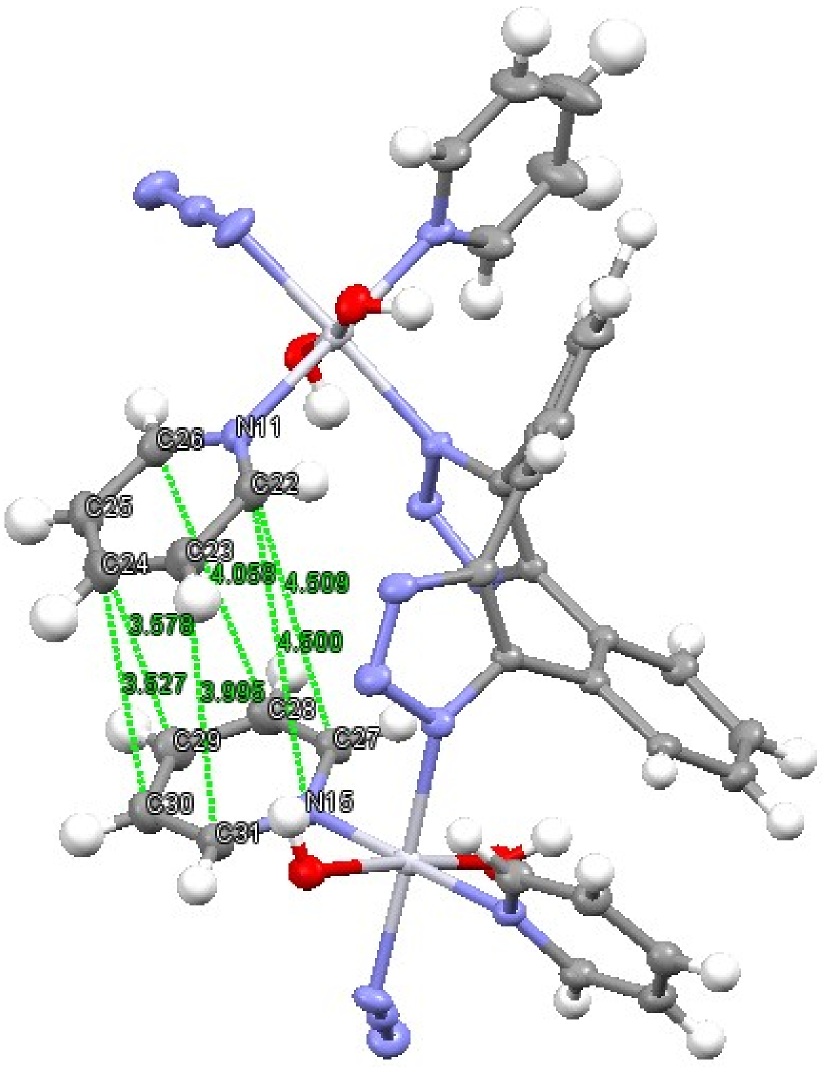

Figure S4. Selected pyridine-pyridine bond distances in X-ray crystallographic structure of **3**-[N1,N3].

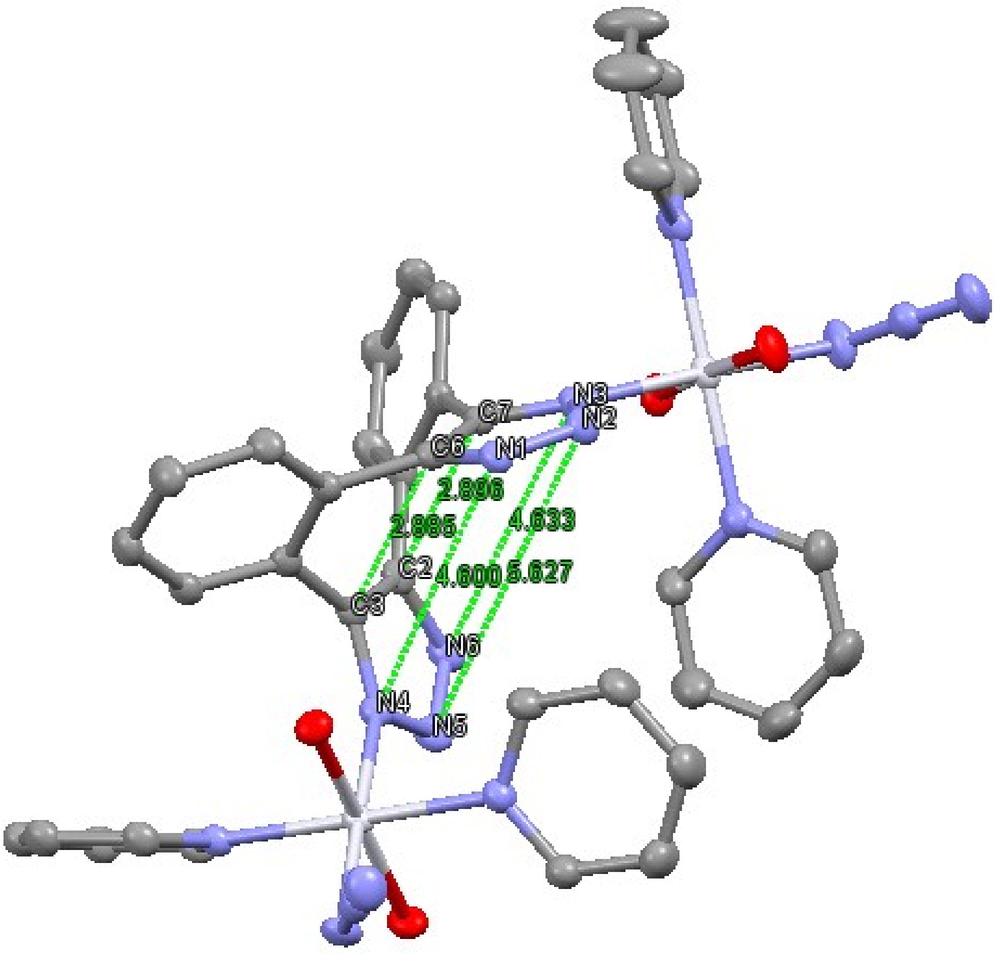

Figure S5. Selected triazole-triazole distances in X-ray crystallographic structure of **3**-[N1,N3].

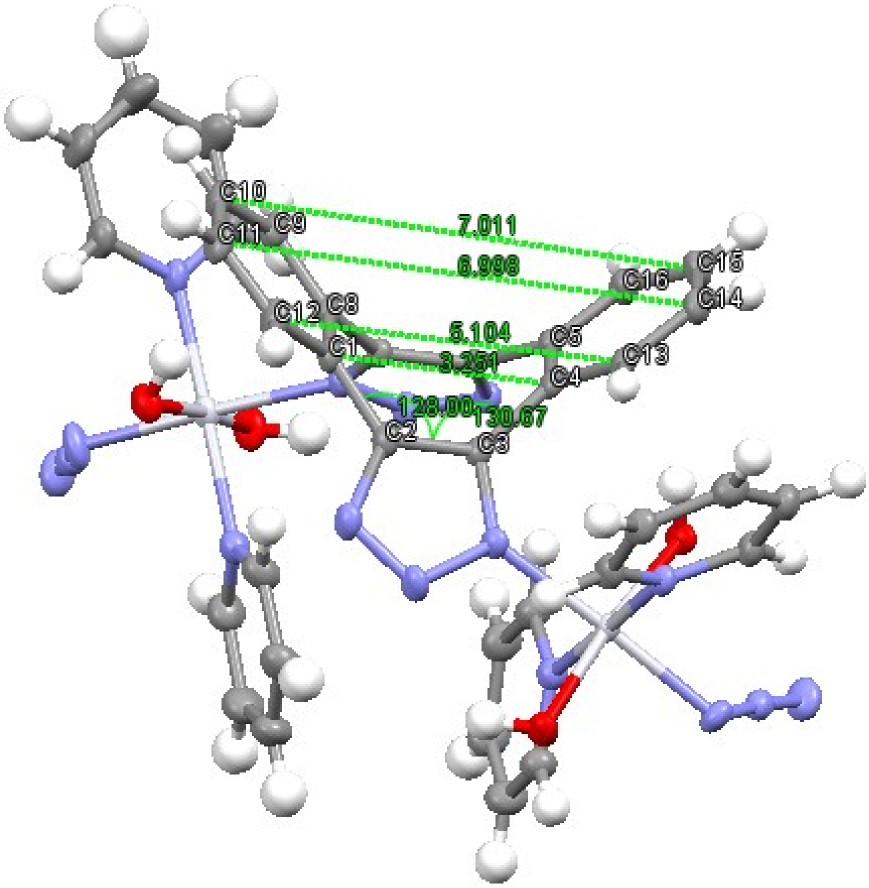

Figure S6. Selected benzene-benzene distances in X-ray crystallographic structure of **3**-[N1,N3].

HRMS

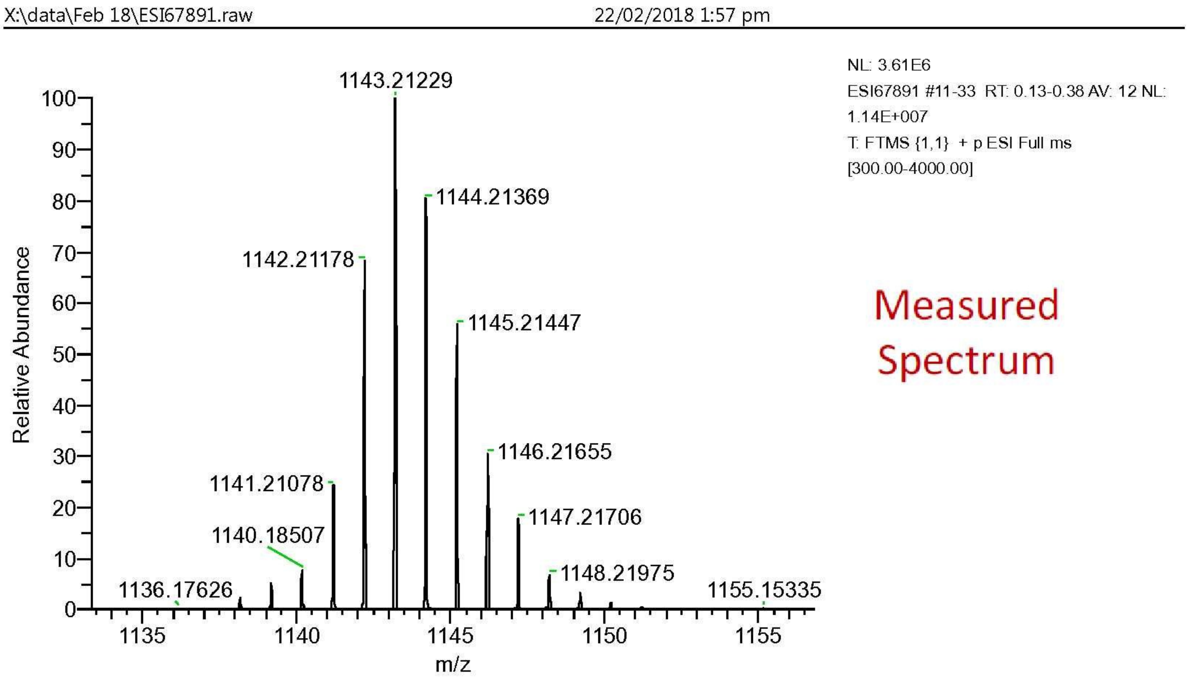

Figure S7. HRMS of [(**3**) + H]$^+$ ($C_{36}H_{32}N_{16}O_4Pt_2H$)*:* 1143.2123 *m/z* found; 1143.2069 *m/z* calc (4.7 ppm error).

MS/MS

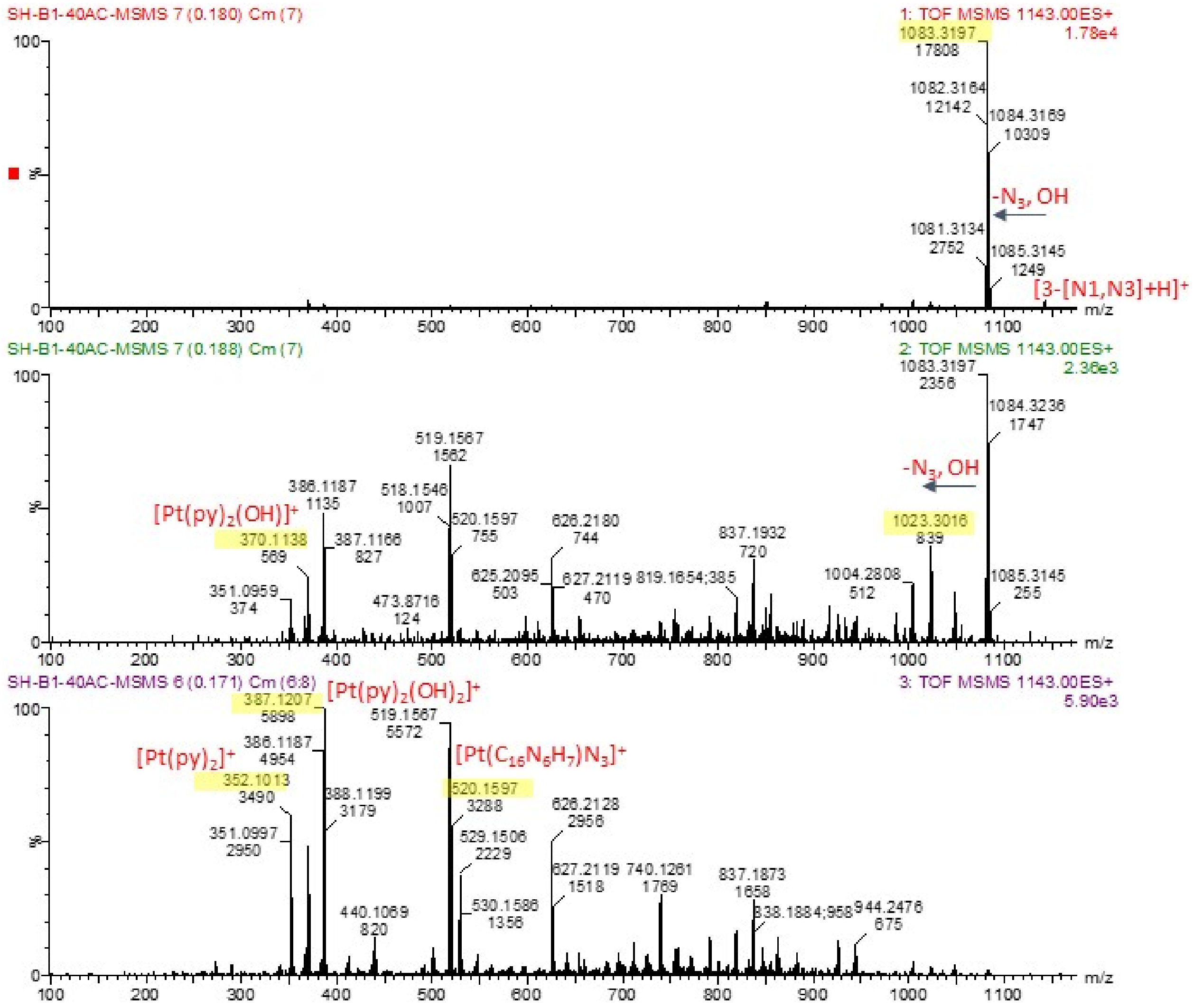

Figure S8. MS/MS (MeCN) of [**3-[N1,N3]** +H]$^+$ (1143 *m/z*) with collision energies of a) 20 eV b) 30 eV c) 40 eV.

NMR spectroscopy

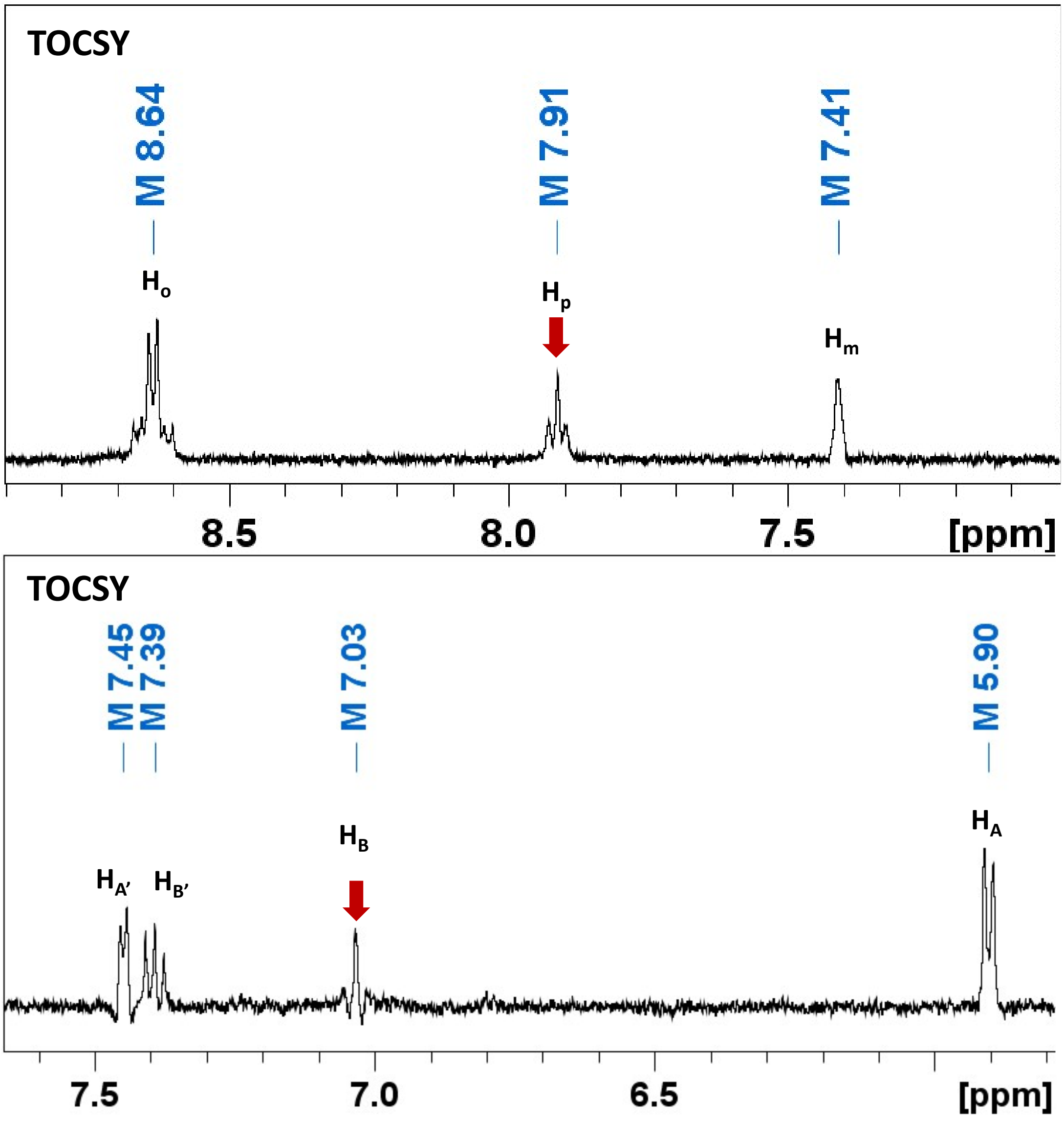

Figure S9. ^{1}H TOCSY NMR spectra of **3**-[**N1,N3**] (d_3-MeCN).

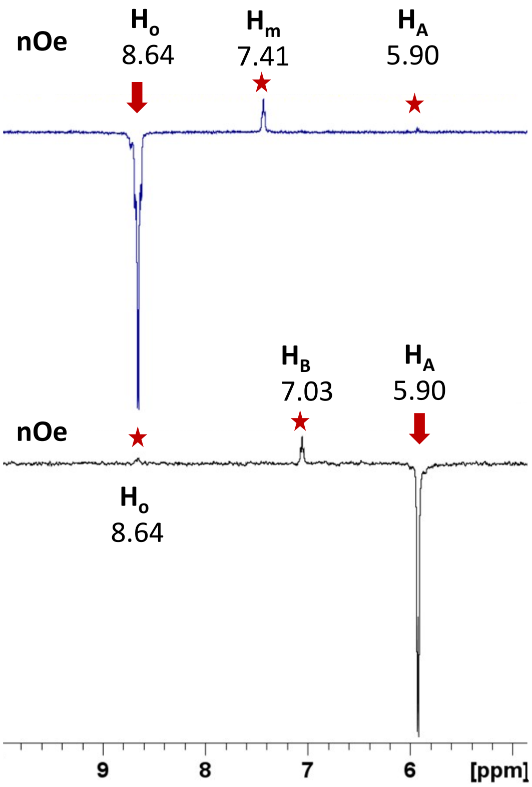

Figure S10. ^{1}H NOESY NMR spectra of **3**-[**N1,N3**] (d_3-MeCN); *top*: 8.64 ppm peak irradiated; *bottom*: 5.90 ppm peak irradiated.

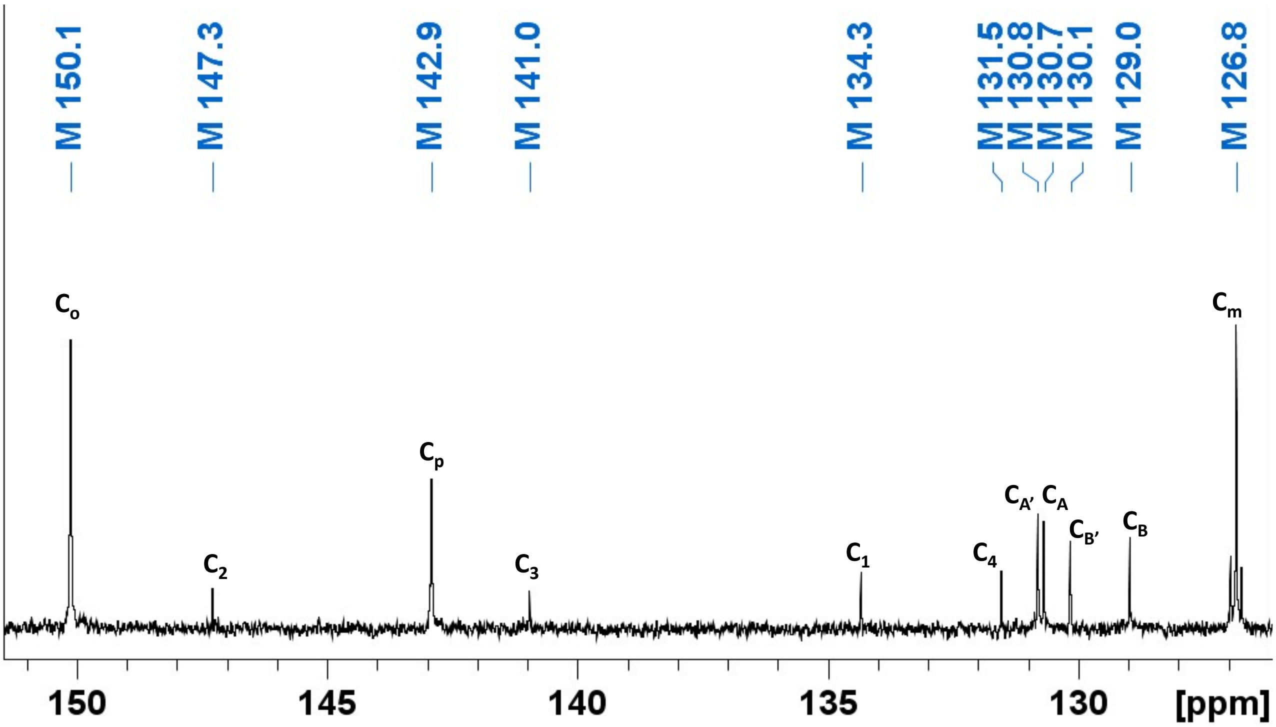

Figure S11. ^{13}C NMR spectrum of **3**-[**N1,N3**] (d_3-MeCN).

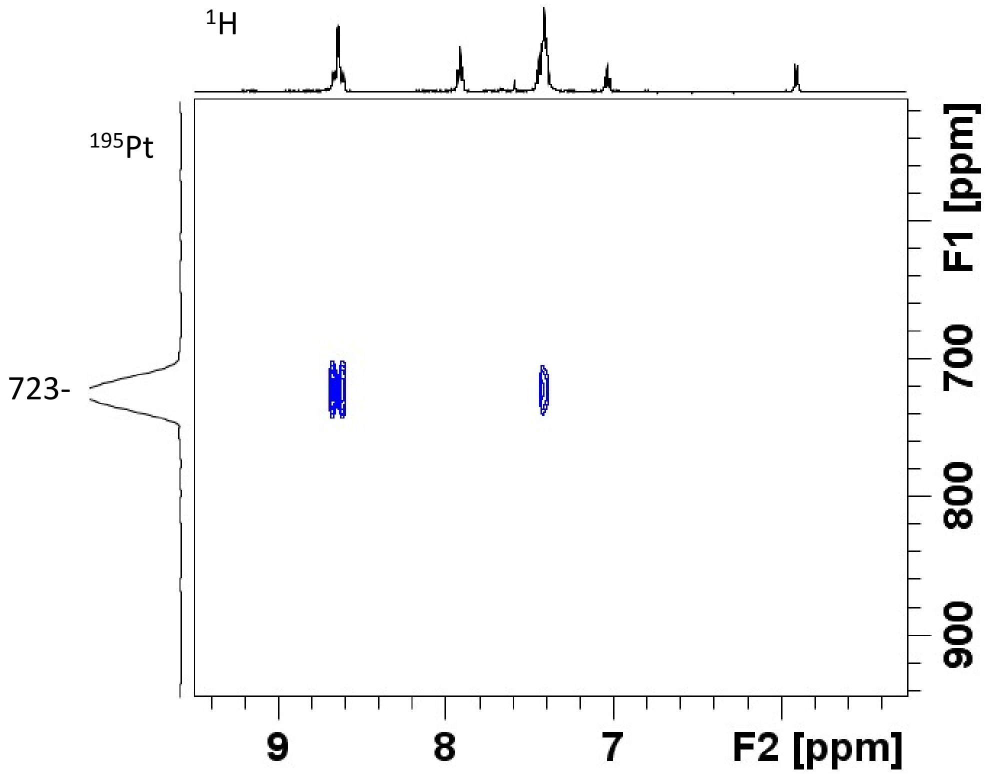

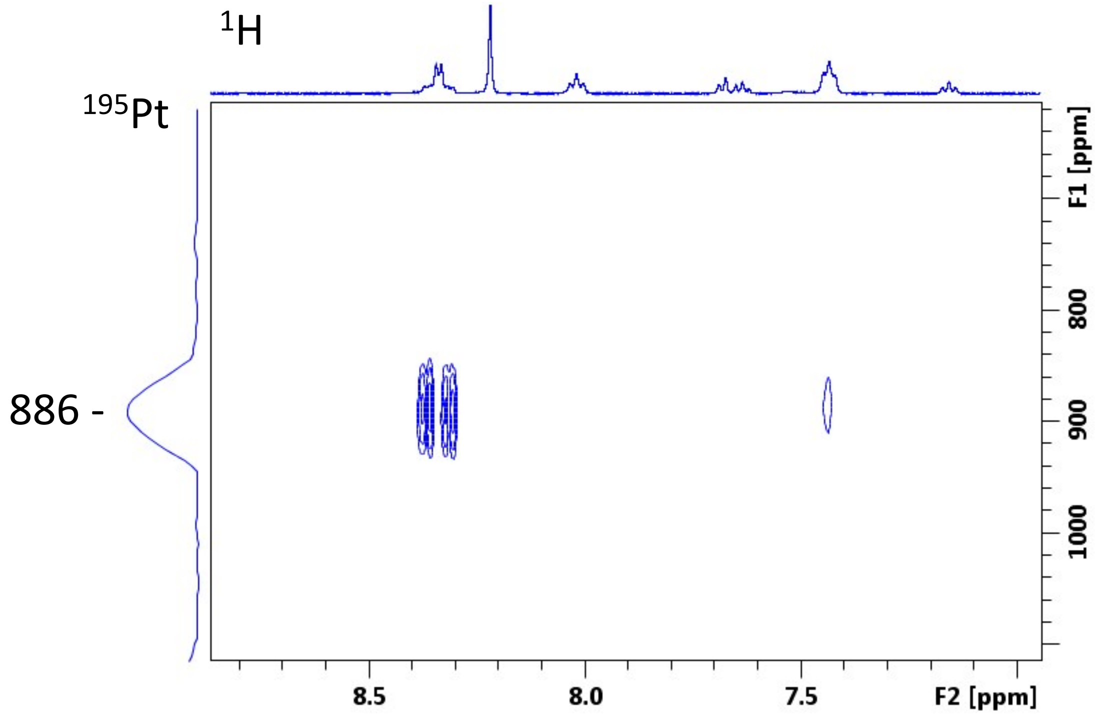

Figure S12. ^{1}H-^{195}Pt HMBC NMR spectrum of **3**-[**N1,N3**]; *top* (d_3-MeCN); *bottom*: with 2 eq. 5'-GMP present (D_2O).

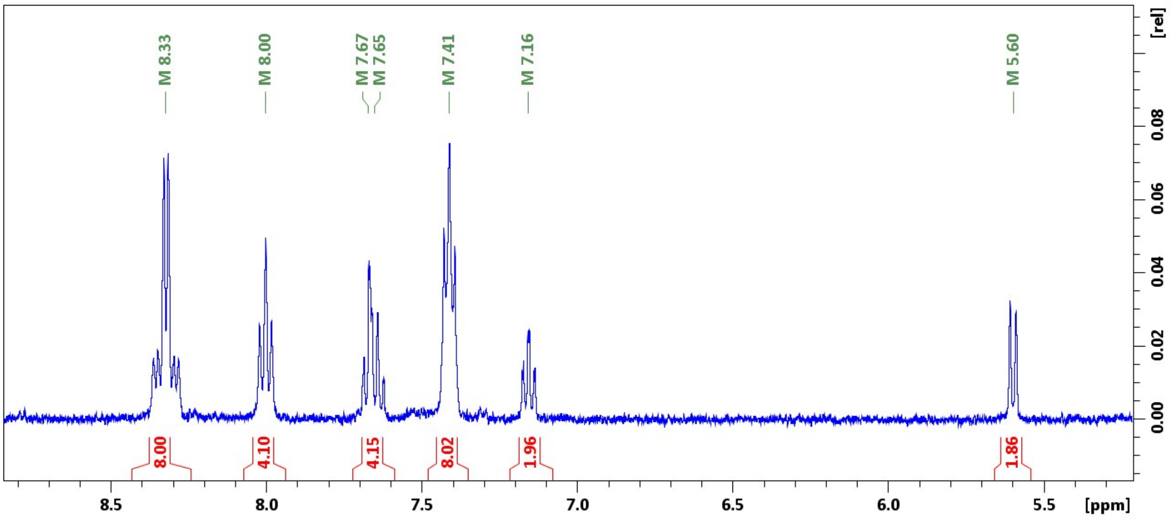

Figure S13. ^1H NMR spectrum of **3**-[**N1,N3**] (D_2O).

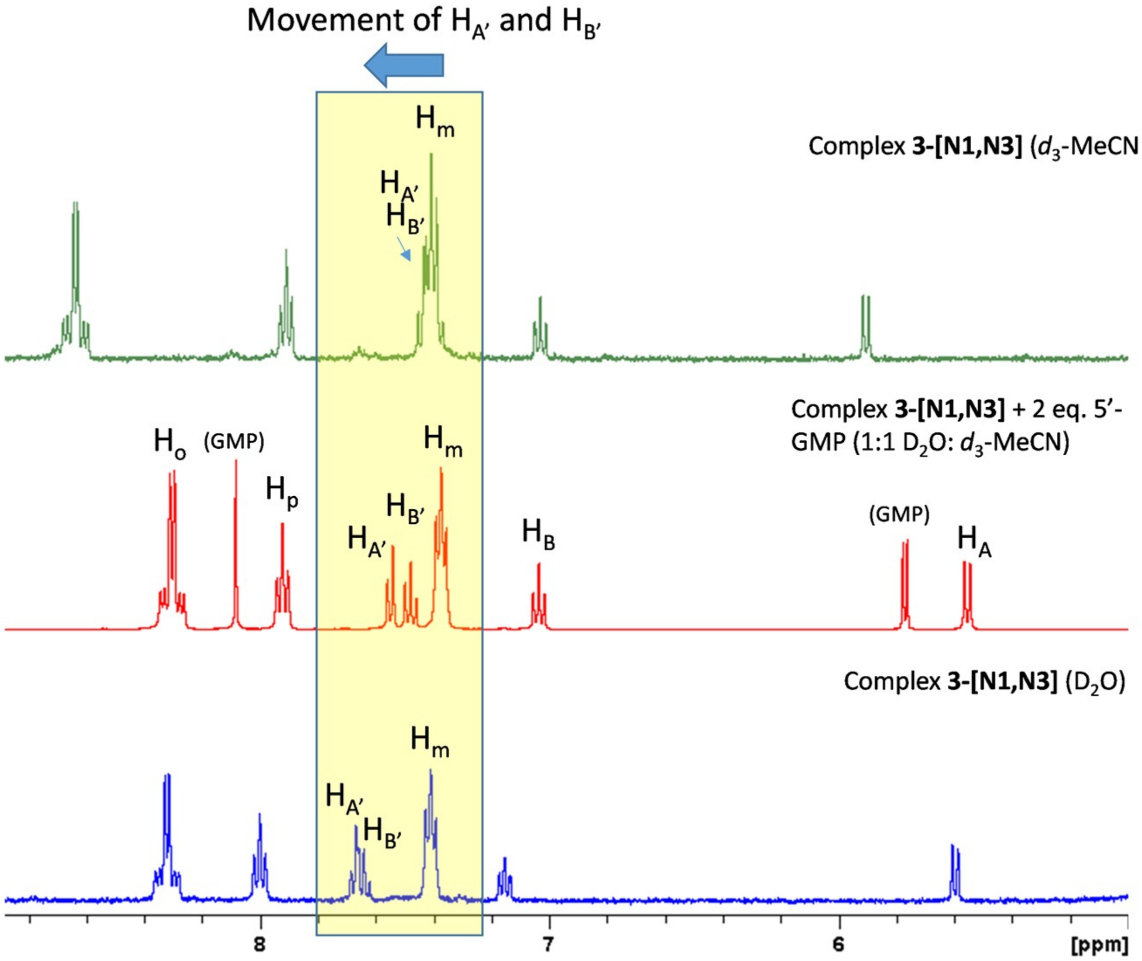

Figure S14. Solvent-dependence of ^1H NMR spectral resonances for **3**-[**N1,N3**]. *Top:* in d_3-MeCN, *middle*: 1:1 v/v D_2O: d_3-MeCN (with 2 eq. 5'-GMP present).; *bottom*: in D_2O.

IR spectrum

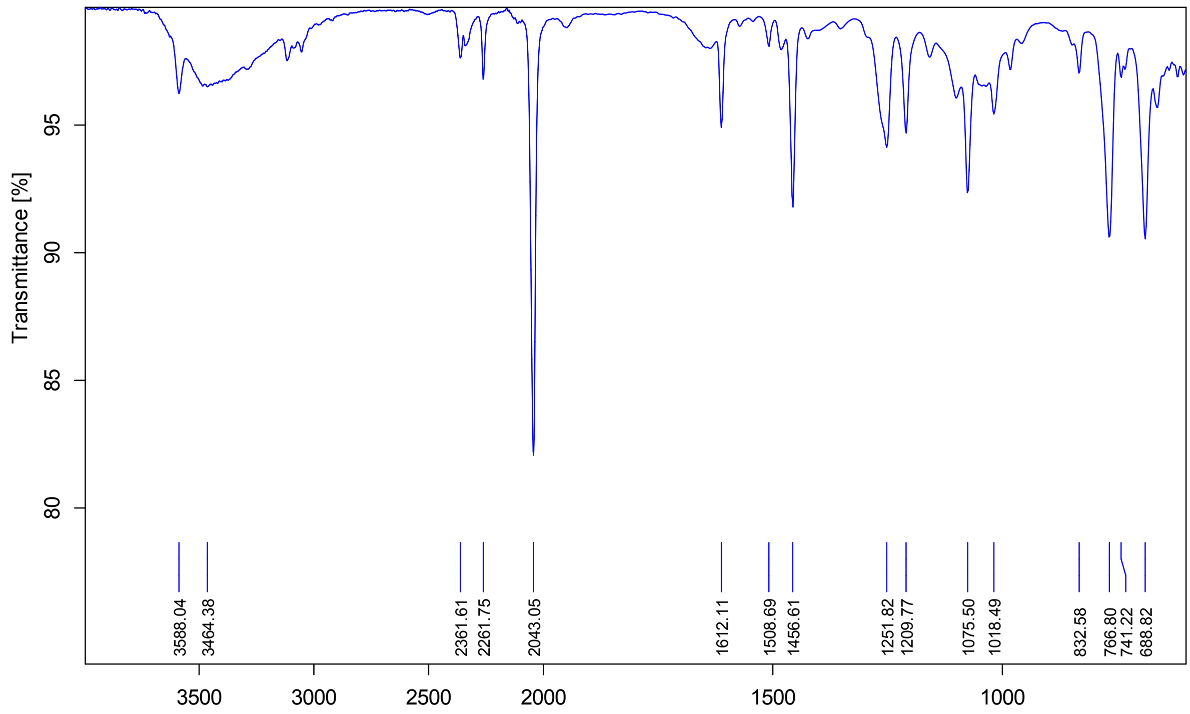

Figure S15. IR spectrum of complex [**3**]-[**N1,N3**] (d_3-MeCN).

UV-Vis Absorbance Spectroscopy

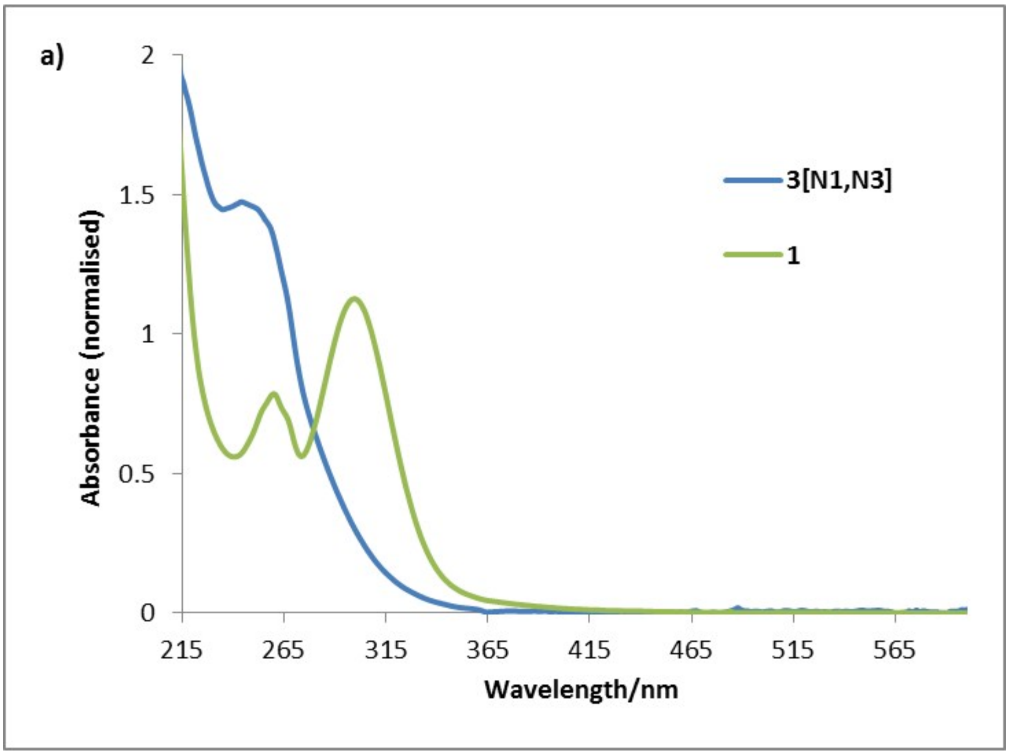

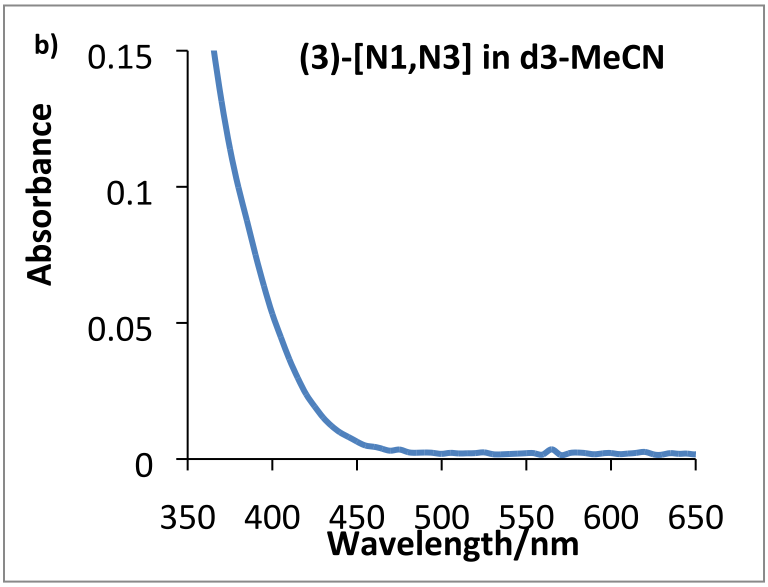

Figure S16; a) UV-Vis spectra of starting material **1** and complex **3-[N1,N3]** (MeCN/H_2O) b) UV-Vis spectrum of complex **3-[N1,N3]** in d_3-MeCN (concentrated sample) showing the limit of absorption at longer wavelengths.

Photochemistry of 3-[N1,N3]

Mass spectrometry

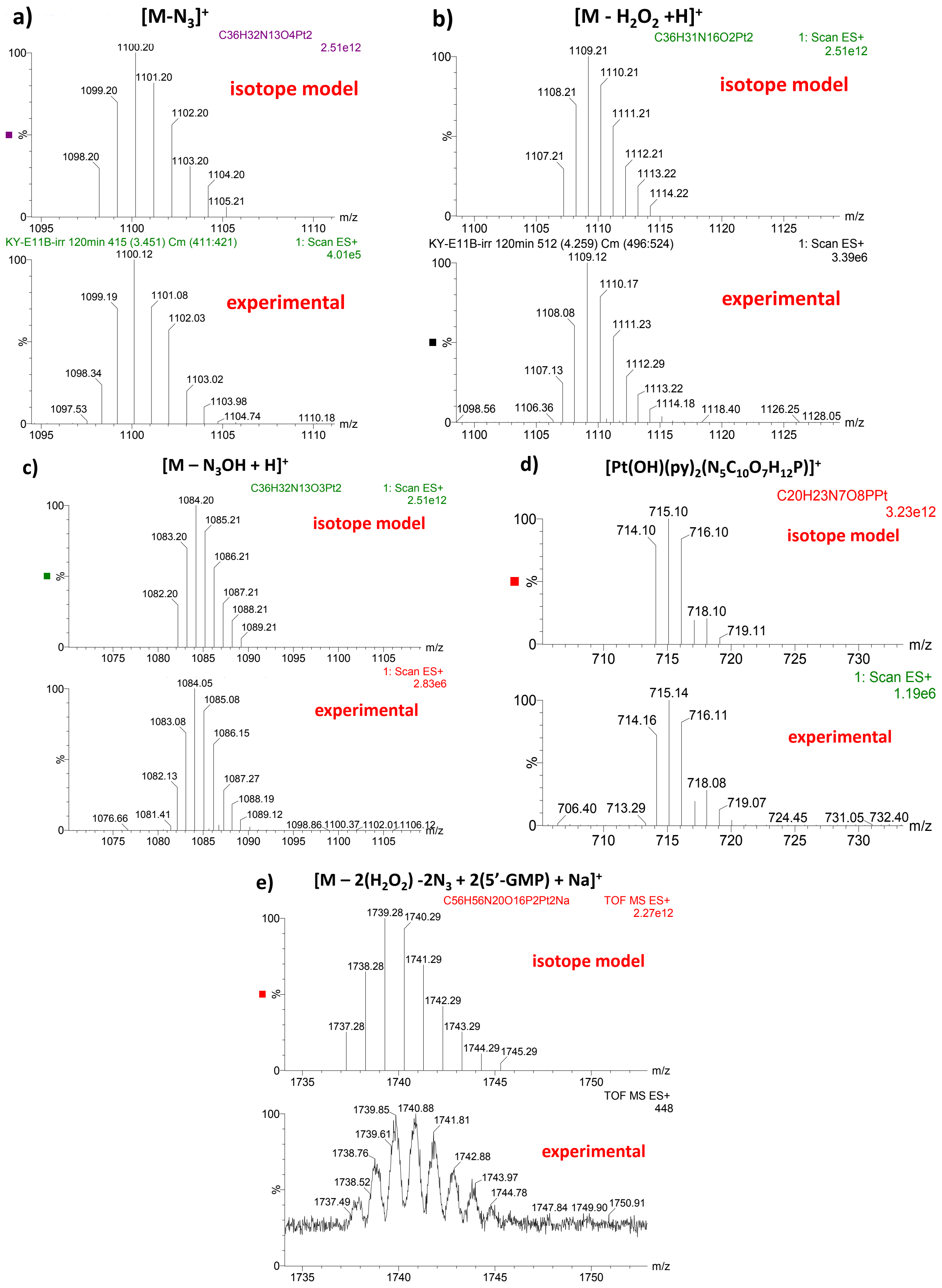

Figure S17. Photoproducts seen following irradiation of **3-[N1,N3]** + 5'-GMP in D_2O (a-d) detected by LCMS in H_2O/MeCN; (e) detected by direct injection into ESI-MS.

NMR Spectroscopy

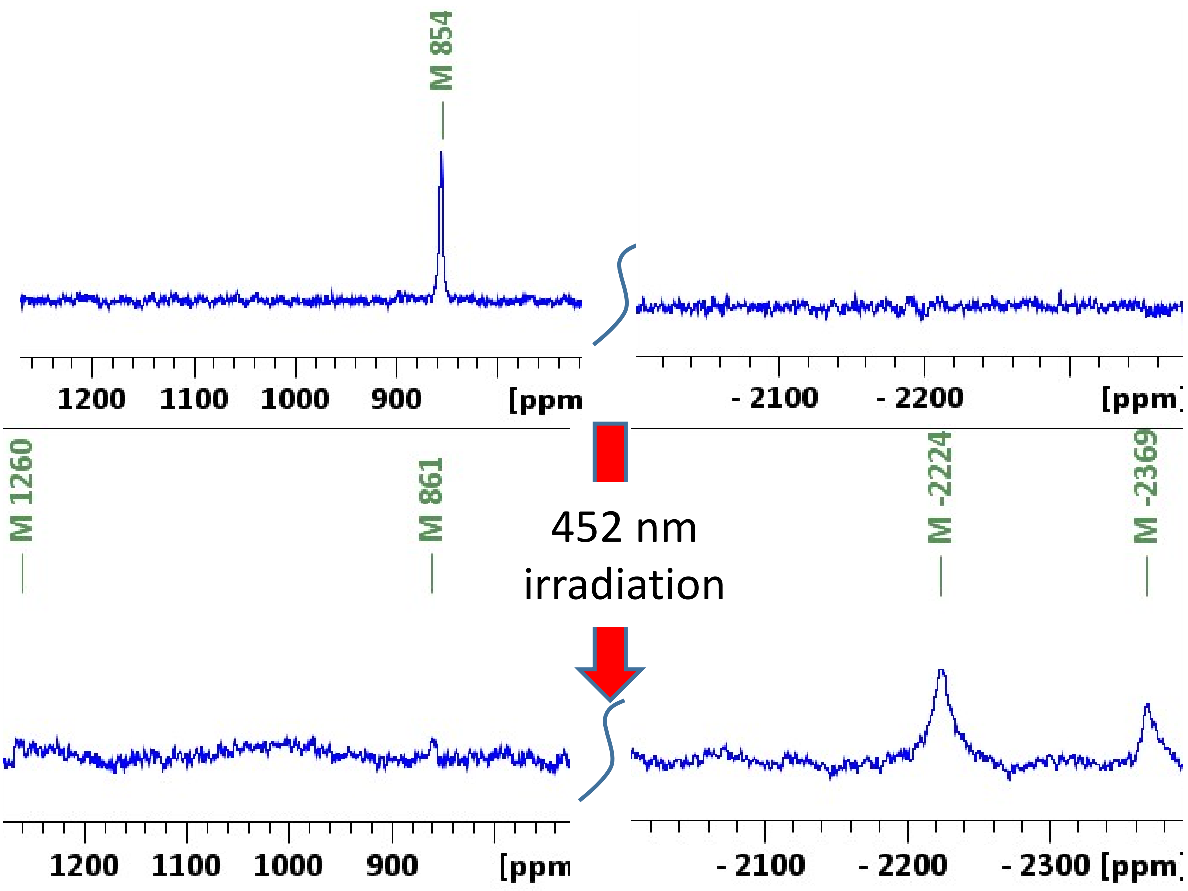

Figure S18. ^{195}Pt NMR spectra (107 MHz, 298 K, 1:1 D_2O:MeCN) of *top*: a solution of 1.5 mM (**3**)-[**N1,N3**] and 2 mol. equiv. of 5'-GMP and *bottom*: showing formation of new Pt^{IV} and Pt^{II} species following irradiation (452 nm, 5 mW, 180 min).

a)

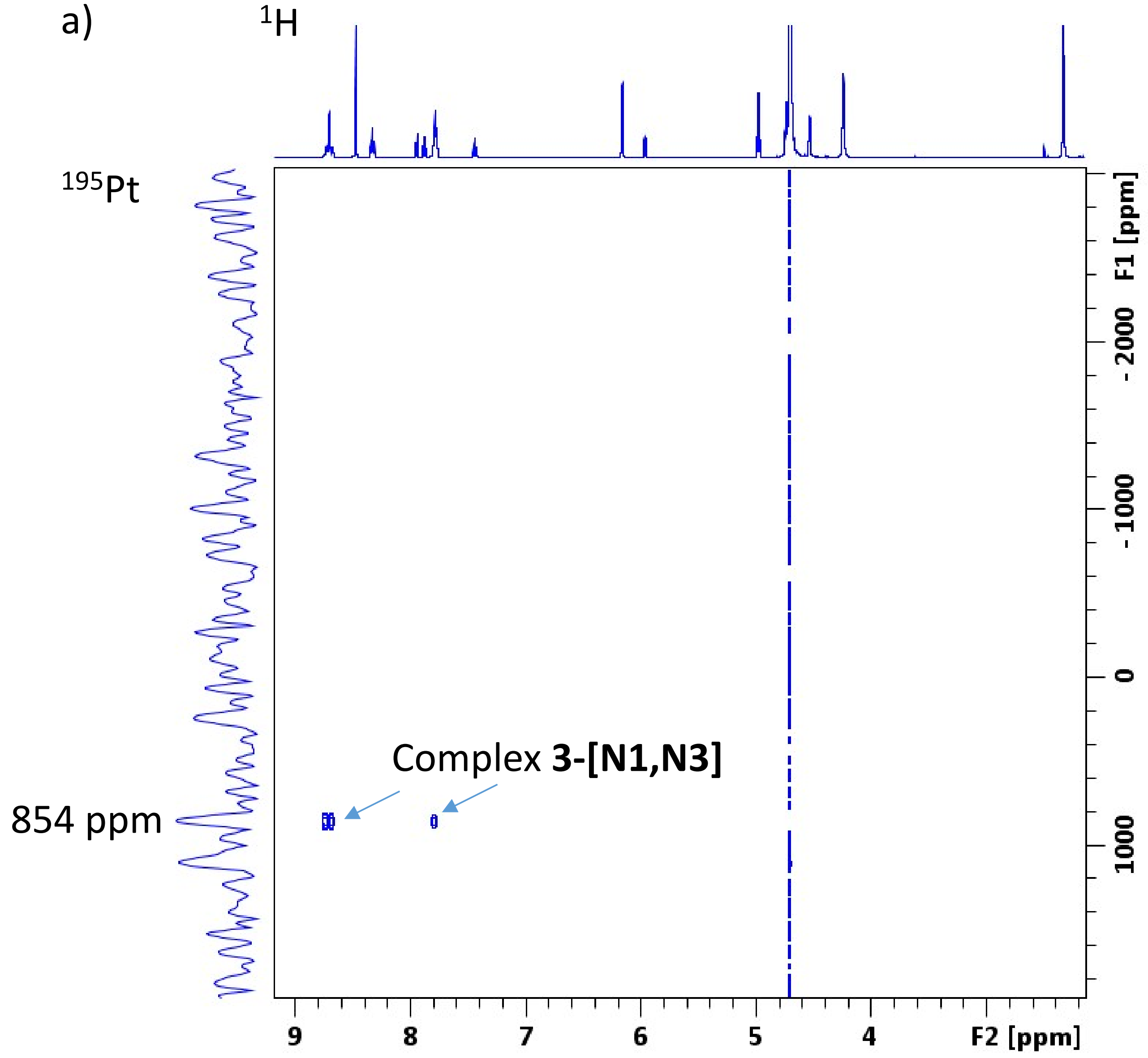

^{1}H
^{195}Pt
854 ppm
Complex **3-[N1,N3]**
F1 [ppm]
-2000
-1000
0
1000
9
8
7
6
5
4
F2 [ppm]

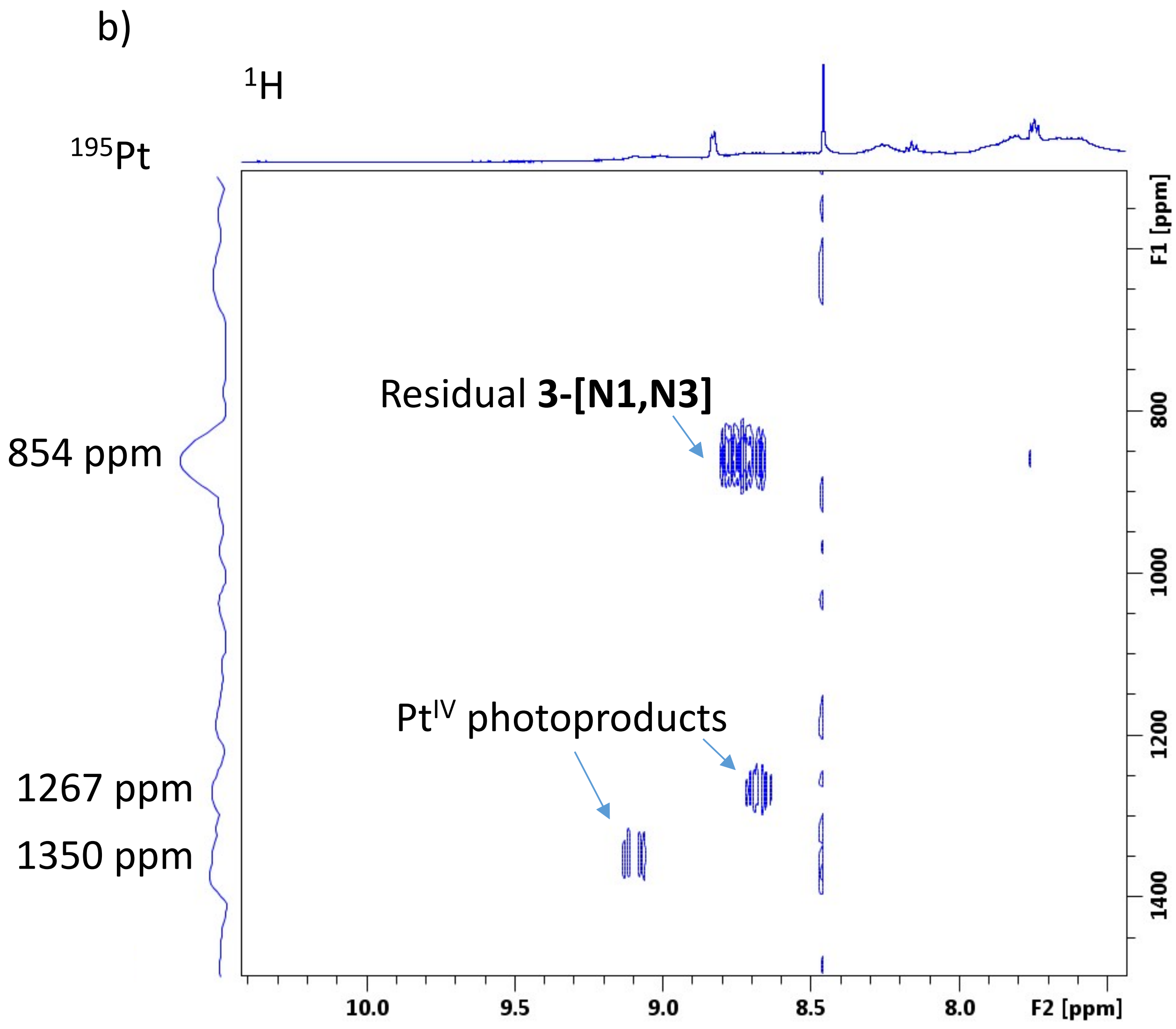

Figure S19. ^{1}H-^{195}Pt HMBC NMR spectrum of **3**-[**N1,N3**] + 5'-GMP (1:1 D_2O: d_3-MeCN) (a) before irradiation and (b) after 180 min irradiation, showing formation of new Pt^{IV} photoproducts in the Pt^{IV} region. The Pt^{II} photoproducts which were observed in Figure S17 were not detected by the wide-sweep ^{1}H-^{195}Pt HMBC, suggested to be due to the rapid relaxation of the Pt^{II} species on the timescale of the HMBC experiment.

EPR Spectroscopy

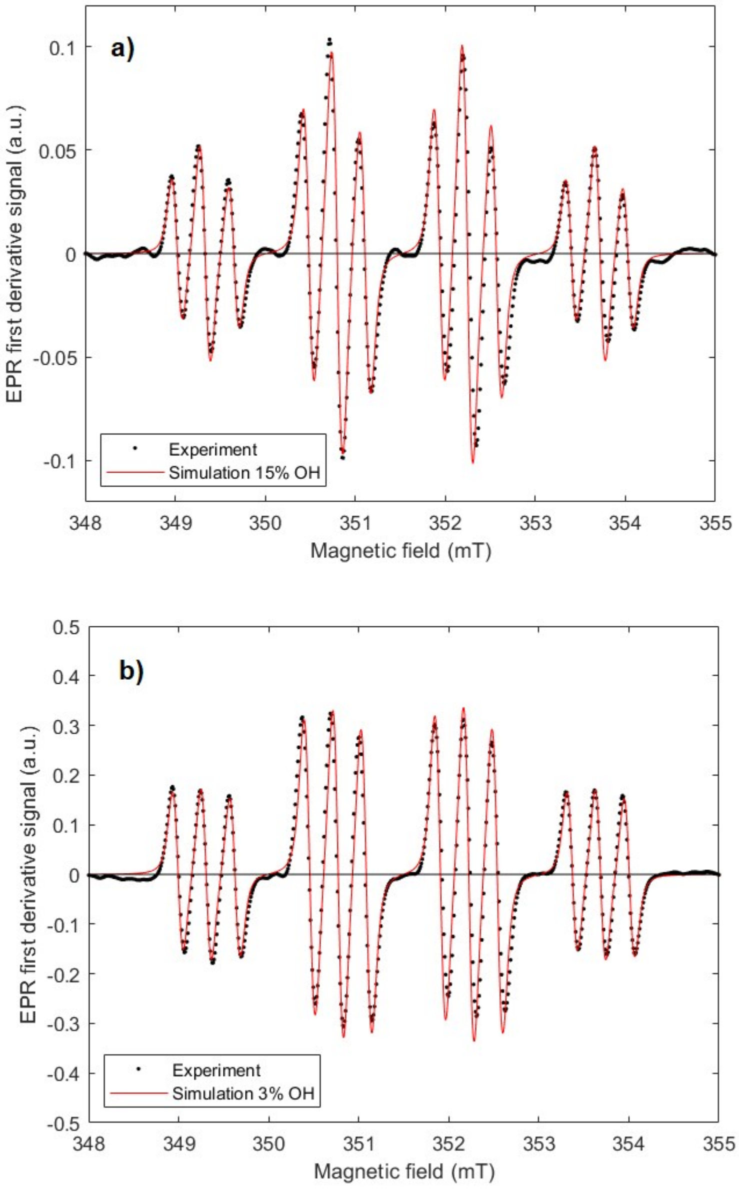

Figure S20. X-band cw-EPR spectra of aqueous solutions of (a) complex **3-[N1,N3]** (2.3 mM) and DMPO (20mM) and (b) **1** (2.3 mM) and DMPO (20 mM) during irradiation (440 – 480 nm), averaged for 1 h (a) and 10 min (b). The molar ratio $N_3^•$:$OH^•$ of trapped radicals is determined from the simulation.

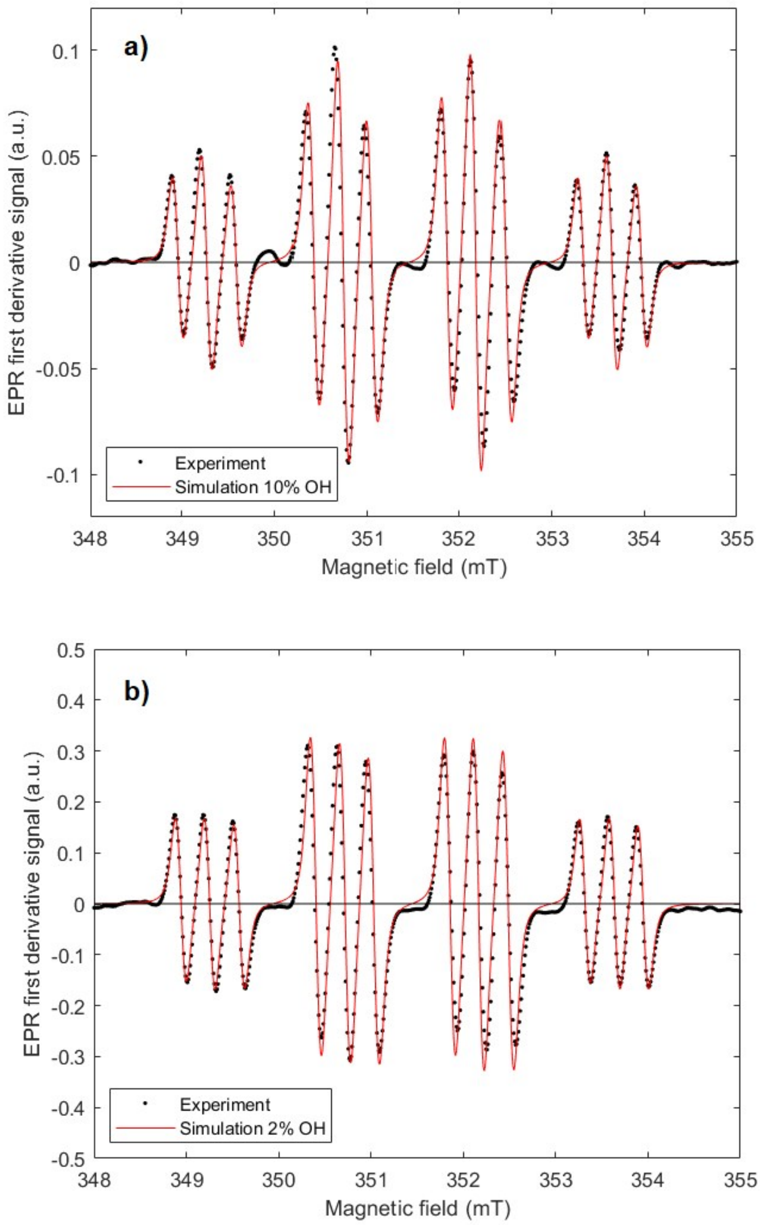

Figure S21. X-band cw-EPR spectra of freshly prepared KNS42 (glioma) cell-free lysate solutions of (a) complex **3-[N1,N3]** (2.3 mM) and DMPO (20mM) and (b) **1** (2.3 mM) and DMPO (20 mM) during irradiation (440 – 480 nm), averaged for 1 h (a) and 10 min (b). The molar ratio $N_3^{\bullet}$:$OH^{\bullet}$ of trapped radicals is determined from the simulation.

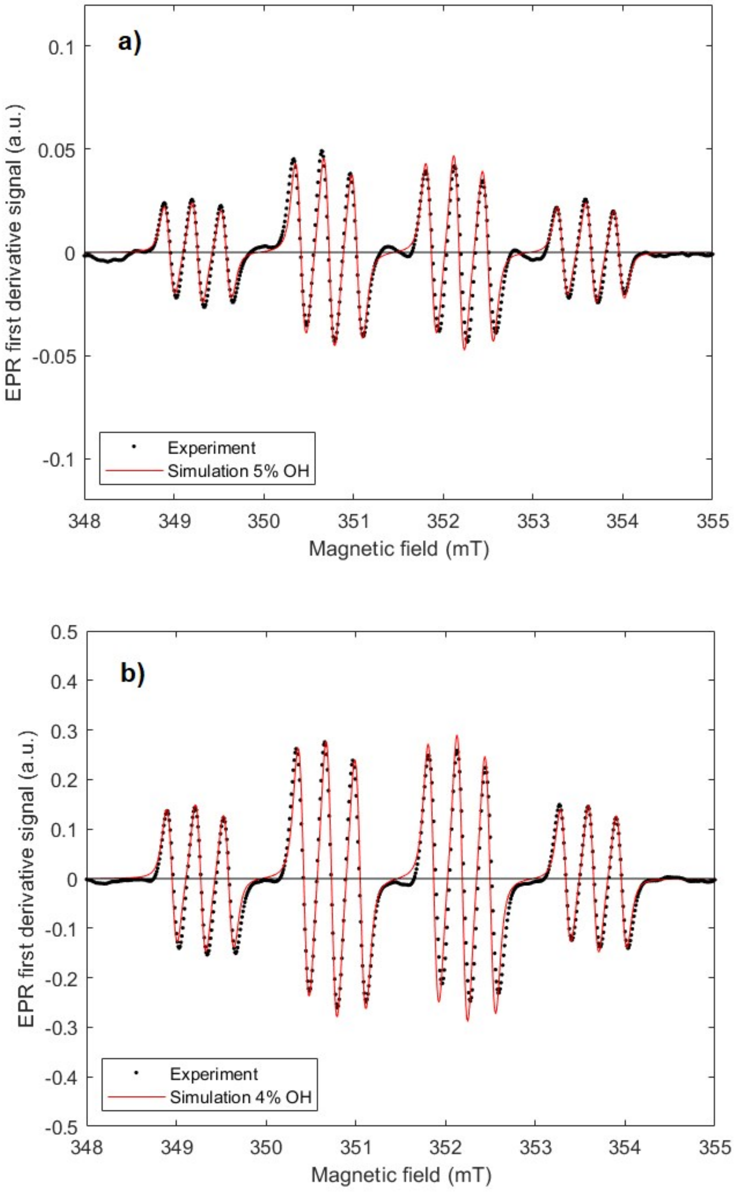

Figure S22. X-band cw-EPR spectra of freshly prepared KNS42 (glioma) cell-free lysate solutions of (a) complex **3-[N1,N3**] (2.3 mM), 5'-GMP (2.3 mM) and DMPO (20 mM) and (b) **1** (2.3 mM), 5'-GMP (2.3 mM) and DMPO (20 mM) during irradiation (440 – 480 nm), averaged for 1 h (a) and 10 min (b). The molar ratio $N_3^{\bullet}$:$OH^{\bullet}$ of trapped radicals is determined from the simulation.

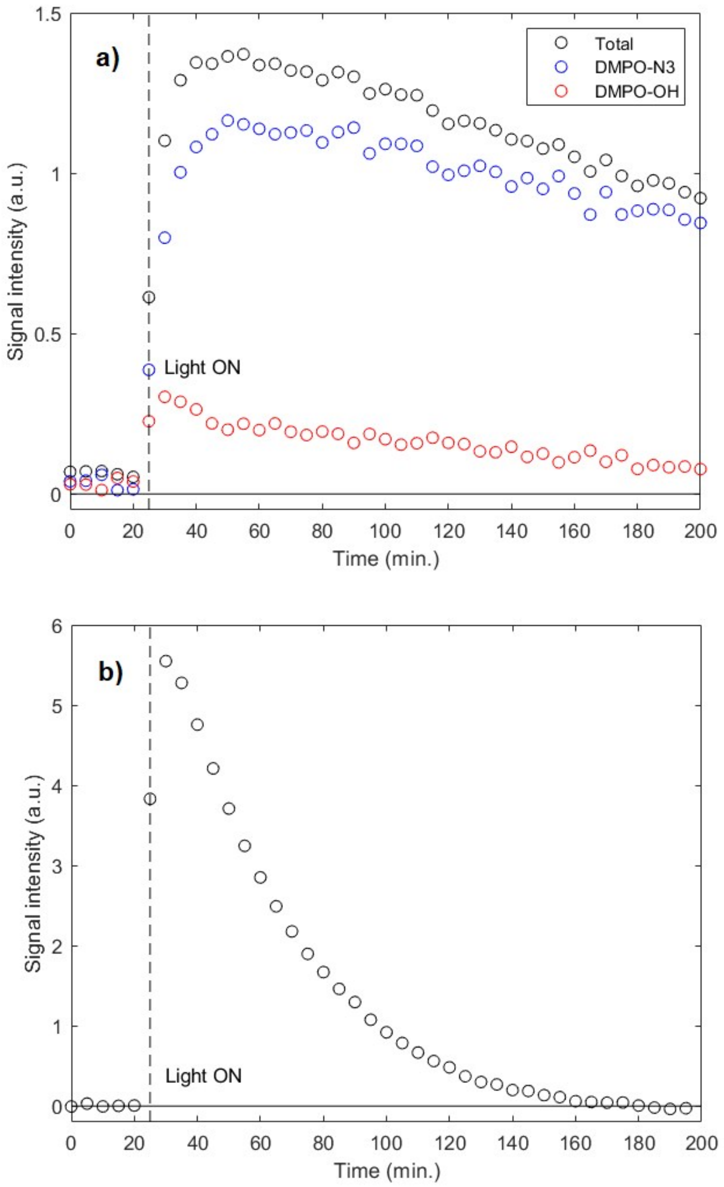

Figure S23. Kinetic profiles for the signals of the radical species trapped in aqueous solutions of (a) complex **3-[N1,N3]** (2.3 mM) and DMPO (20 mM) and (b) **1** (2.3 mM) and DMPO (20 mM) during irradiation (440 – 480 nm). Spectrum (b) only shows the total radical signal intensity, as the amount of DMPO-OH determined from the simulations was very small.

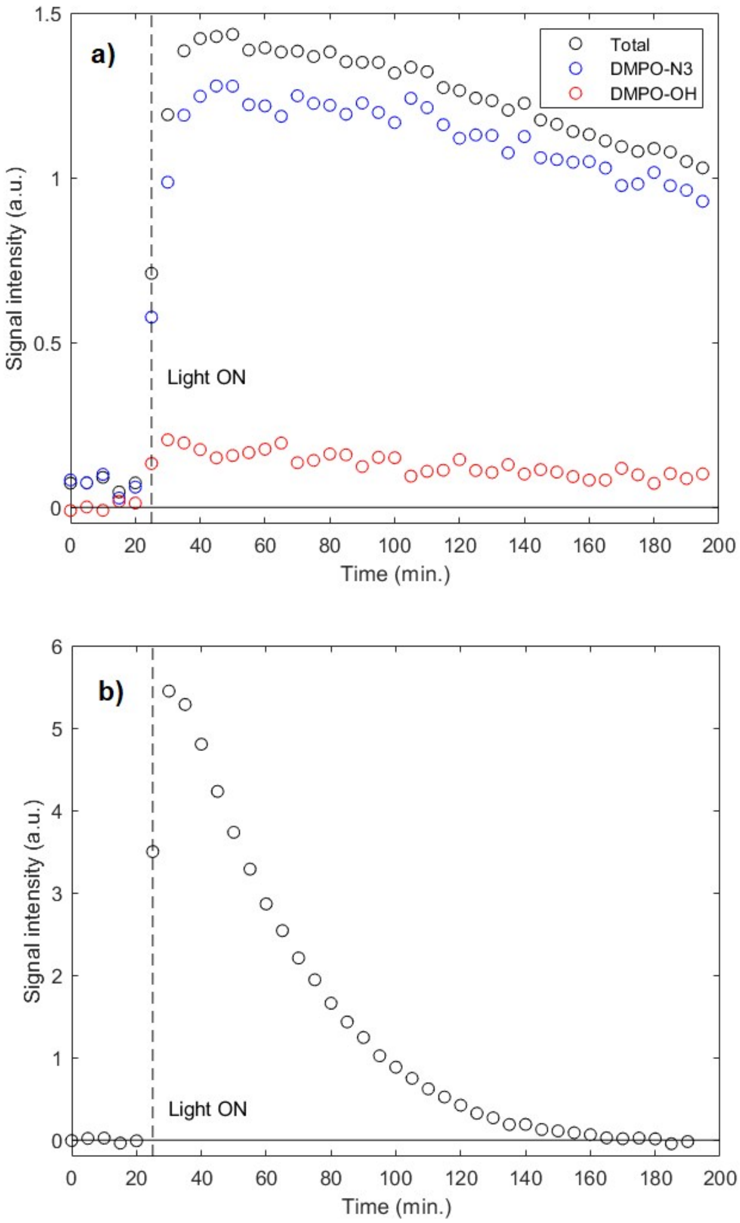

Figure S24. Kinetic profiles for the signals of the radical species trapped in freshly prepared KNS42 (glioma) cell-free lysate solutions of (a) complex **3-[N1,N3]** (2.3 mM) and DMPO (20 mM) and (b) **1** (2.3 mM) and DMPO (20 mM) during irradiation (440 – 480 nm). Spectrum (b) only shows the total radical signal intensity, as the amount of DMPO-OH determined from the simulations was very small.

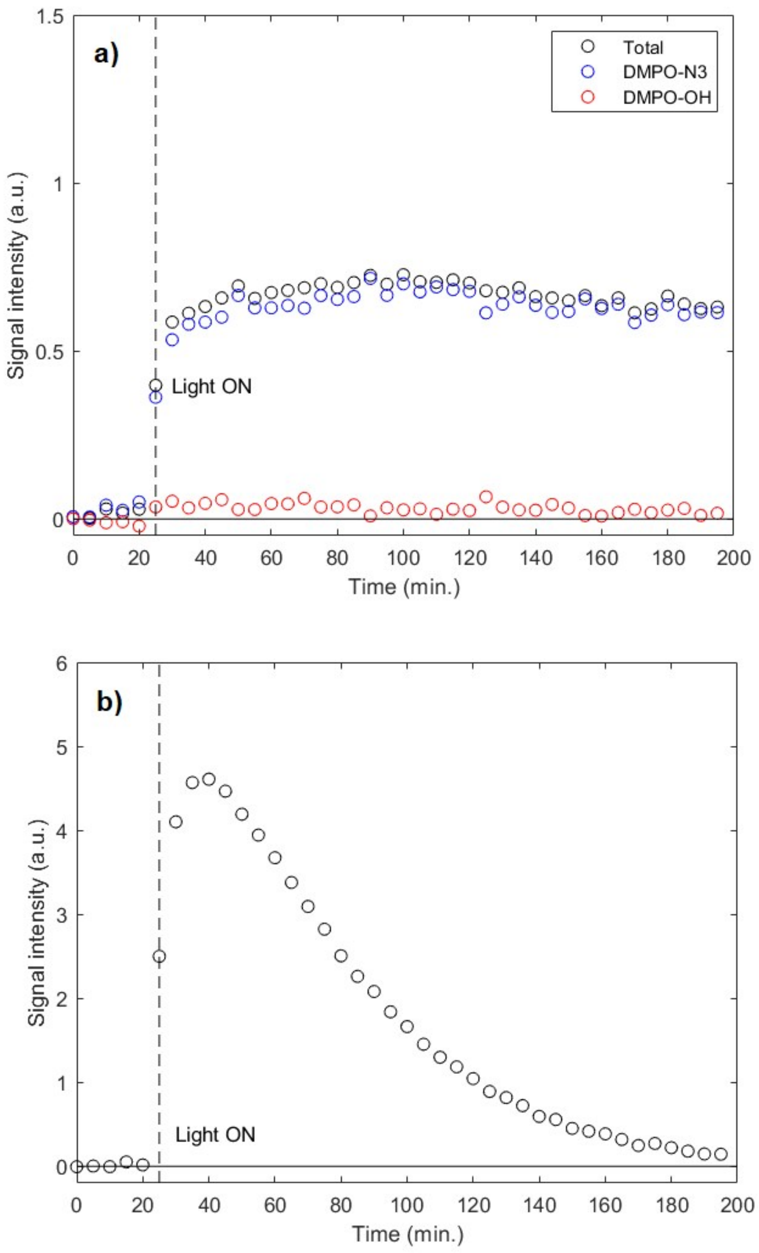

Figure S25. Kinetic profiles for the signals of the radical species trapped in freshly prepared KNS42 (glioma) cell-free lysate solutions of (a) complex **3-[N1,N3**] (2.3 mM), 5'-GMP (2.3 mM) and DMPO (20mM) and (b) **1** (2.3 mM), 5'-GMP (2.3 mM) and DMPO (20 mM) during irradiation (440 – 480 nm). Spectrum (b) only shows the total radical signal intensity, as the amount of DMPO-OH determined from the simulations was very small.

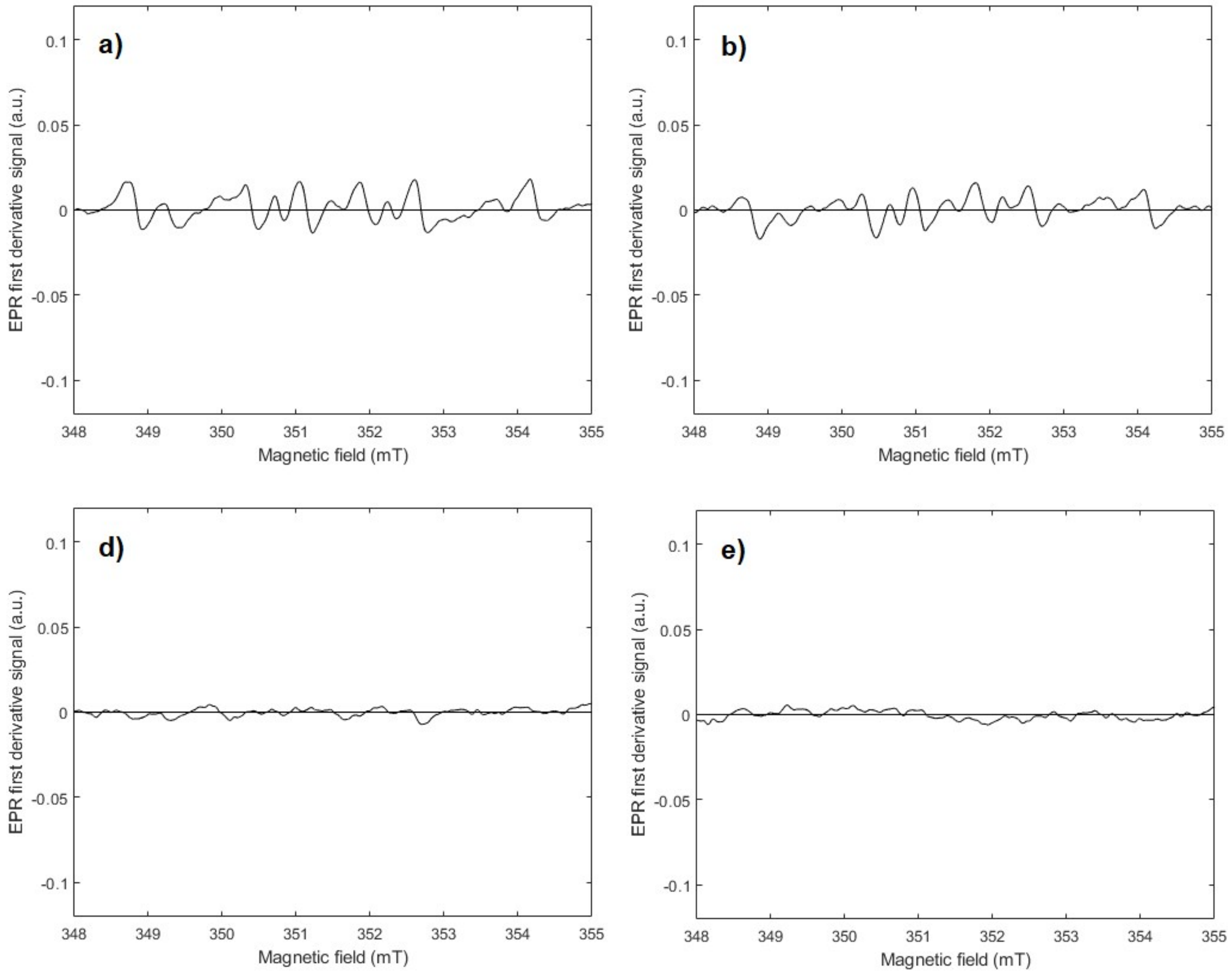

a)
b)
d)
e)
EPR first derivative signal (a.u.)
Magnetic field (mT)
0.1
0.05
0
-0.05
-0.1
348
349
350
351
352
353
354
355

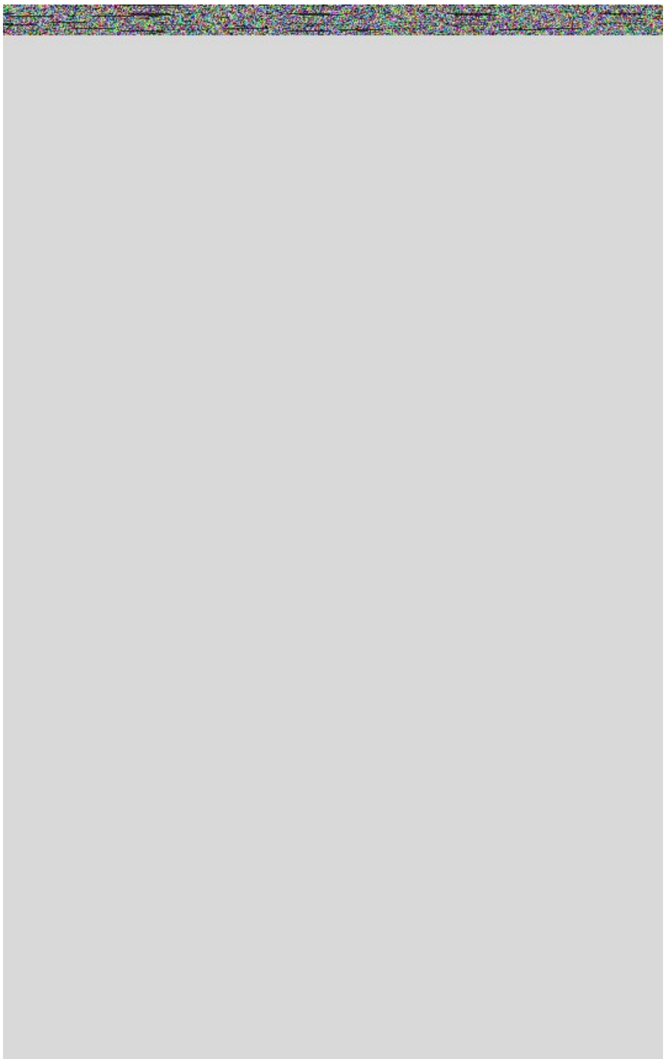

Figure S26. X-band cw-EPR spectra of complex **3 [N1,N3**] + DMPO (a, b and c) and the starting material **1** + DMPO (d, e and f) in water (a, d), freshly prepared KNS42 (glioma) cell-free lysate (b, e) and lysate + 2.3 mM 5’-GMP, in the dark. For all measurements the concentrations were 2.3 mM Pt and 20 mM DMPO. The spectra were averaged for 25 min.

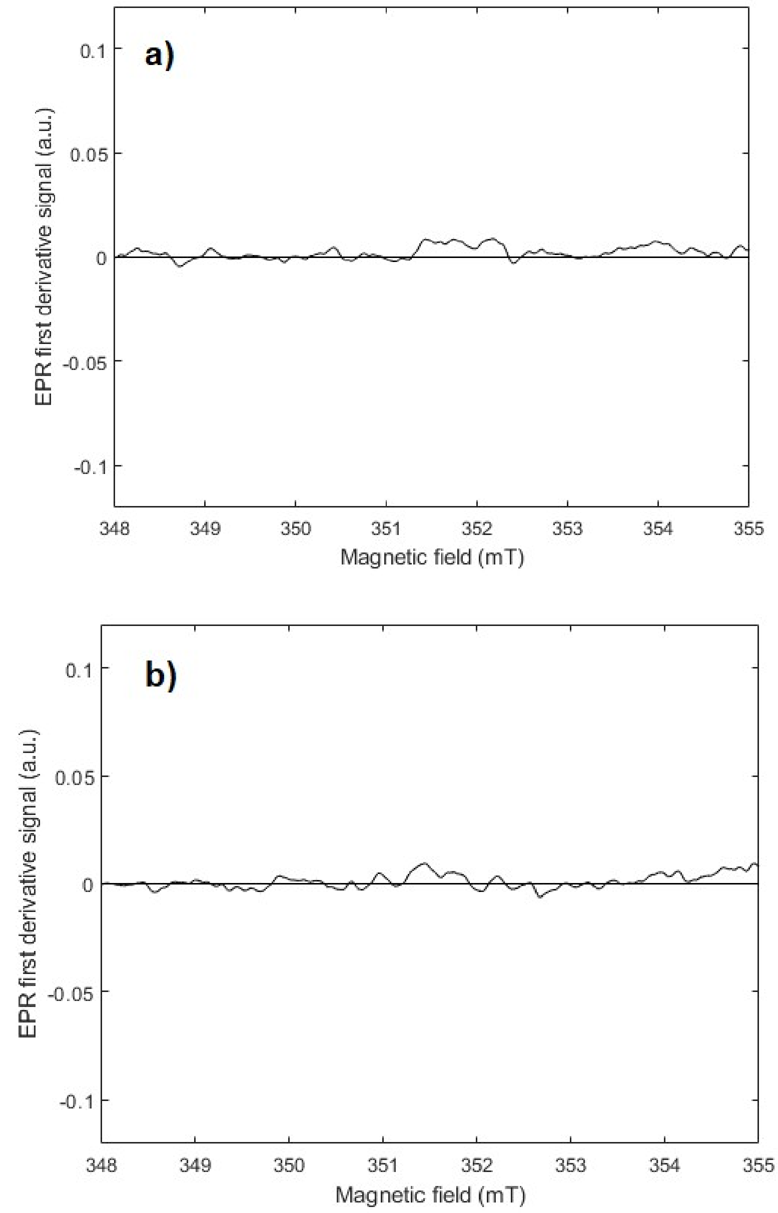

Figure S27. X-band cw-EPR spectra of (a) 20 mM DMPO in KNS42 lysate and (b) 2.3 mM 5'-GMP + 20 mM DMPO in cell-free lysate, both under continuous illumination for 25 min.

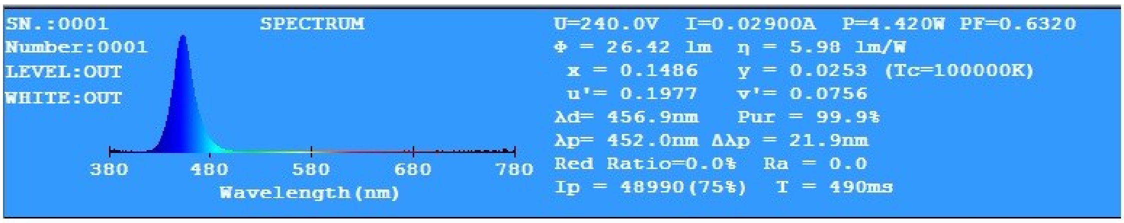

Figure S28. Spectral output of MiniSun (GU10 27 SMD) blue LED bulb, λ_{max} = 452 nm.

Photochemistry discussion

Irradiation of **3**-[N1,N3] with 452 nm light resulted in the formation of new Pt^{IV} and Pt^{II} species, as demonstrated by ^{195}Pt NMR spectroscopy. Radical formation ($N_3^{\bullet}$, $OH^{\bullet}$) is likely to occur when a Pt^{IV} centre in **3**-[N1,N3] undergoes photoreduction. For elimination of two radicals from one Pt^{IV} centre, we suggest that this involves two stepwise one-electron donations from azido and hydroxyl radical - or two hydroxyl radicals, depending on the radicals generated - to the Pt centre.[10] The formation of a Pt^{III} centre is a plausible intermediate, such species having previously been detected by ESI-MS of photoactivated Pt^{IV} prodrugs by ourselves[11] and by ESI-MS collision-induced dissociation of Pt^{IV} prodrugs by others.[12] Detailed experimental and theoretical investigations of the electrochemical reduction of Pt^{IV} to Pt^{II} have also determined that electron transfer and Pt−L bond cleavage occurs in a stepwise fashion, with a metastable six-coordinate Pt(III) intermediate formed upon addition of a single electron; the loss of both axial ligands is then associated with the second electron transfer.[13] Although Pt^{III} species are likely to be transient intermediates following the ejection of either an azido or a hydroxyl radical, closed-shell Pt^{II} or Pt^{IV} centres are significantly more stable than Pt^{III}; the latter typically only being stable in the presence of suitable bulky ligands over which the Pt^{III} unpaired electron can be delocalised.[14] We suggest that for species where two ligands have been photoejected, that this is more likely to occur from the same Pt^{IV} centre, forming a Pt^{IV}-Pt^{II} photoproduct, as opposed to a Pt^{III}- Pt^{III} species.

Of the photoproducts observed by ESI-MS, those which could be assigned as consistent with observed masses are shown below, with suggested formulae and putative oxidation states:

[**3**-[**N1,N3**] – $N_3]^+$ at 1110.12 *m/z;* $[Pt^{IV}(N_3)(Py)_2(OH)_2(C_{16}H_8N_6)Pt^{IV}(Py)_2(OH)_2]^+$, predicted $C_{36}H_{32}N_{13}O_4Pt_2$, 1110.20 *m/z.*

This formally charged complex is formed through loss of azide anion (N_3^-). It is likely that in aqueous solution that this species is hydrated in the vacant coordination site thus: $[Pt^{IV}(N_3)(Py)_2(OH)_2(C_{16}H_8N_6)Pt^{IV}(Py)_2(OH)_2(OH_2)]^+$, with an appropriate counterion.

[**3**-[**N1,N3**] - H_2O_2 +H$]^+$at 1109.12 *m/z*; $[Pt^{II}(N_3)(Py)_2(C_{16}H_8N_6)Pt^{IV}(N_3)(Py)_2(OH)_2 + H]^+$, predicted $C_{36}H_{31}N_{16}O_2Pt_2$, 1109.21 *m/z*.

[**3**-[**N1,N3**] – N_3OH + $H^+]^+$ 1084.05 *m/z*; $[Pt^{II}(OH)(Py)_2(C_{16}H_8N_6)Pt^{IV}(N_3)(Py)_2(OH)_2 + H]^+$, predicted $C_{36}H_{32}N_{13}O_3Pt_2$, 1084.20 *m/z*.

$[Pt^{II}(OH)(py)_2(N_5C_{10}O_7H_{12}P)]^+$ at 715.14 *m/z;* cyclic-5'-GMP species, predicted $C_{20}H_{23}N_7O_8PPt$, = 715.10 *m/z*. Possible structure:

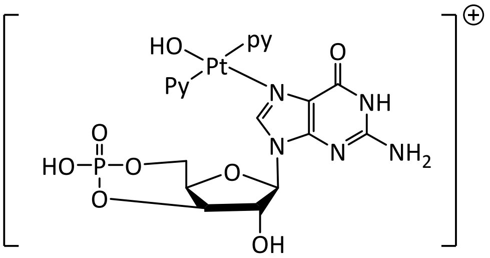

[**3**-[**N1,N3**] – $2(H_2O_2)$ $-2N_3$ + 2(5'-GMP) + $Na]^+$ at 1739.85 *m/z;* Pt^{II}*bis*-GMP adduct: $C_{56}H_{56}N_{20}O_{16}P_2Pt_2Na$, 1739.29 *m/z*. Possible structure:

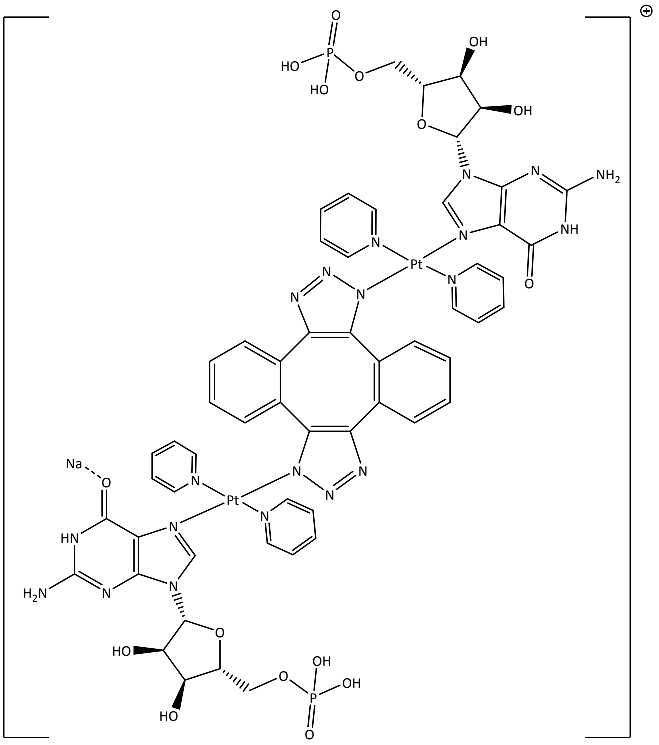

Note: The photoproducts detected by ESI-MS are by necessity either formally charged, or sodiated/protonated. These species therefore represent only a subset of the possible photoproducts – those which are uncharged, or negatively charged not being unobserved. Furthermore, ^{195}Pt NMR spectroscopy is a relatively insensitive slow technique, and unlikely to detect minor – or transient – photoproducts. Isolation and complete structural characterisation of the stable photoproducts (and determining the photochemical pathways, subsequent photochemistry of intermediates *etc.*) is outside the scope of this communication, these more detailed experiments are currently underway.

References

1 A. Bax and M. F. Summers, *J. Am. Chem. Soc.*, 1986, **108**, 2093.

2 S. Stoll and A. Schweiger, *J. Magn. Reson.*, 2006, **178**, 42.

3 N. J. Farrer, J. a. Woods, L. Salassa, Y. Zhao, K. S. Robinson, G. Clarkson, F. S. MacKay and P. J. Sadler, *Angew. Chemie-International Ed.*, 2010, **49**, 8905.

4 H. N. C. Wong, P. Garratt and F. Sondheimer, *J. Am. Chem. Soc.*, 1974, **96**, 5604.

5 A. Orita, D. Hasegawa, T. Nakano and J. Otera, *Chem. - A Eur. J.*, 2002, **8**, 2000.

6 CrysAlisPro, Agilent Technologies, Version 1.171.35.8.

7 G. M. Sheldrick in SHELXL97, Programs for Crystal Structure Analysis (Release 97-2), Institut für Anorganische Chemie der Universität, Tammanstrasse 4, D-3400 Göttingen, Germany, 1998.

8 G. M. Sheldrick, *Acta Crystallogr. Sect. A*, 1990, **46**, 467.

9 G. M. Sheldrick, *Acta Crystallogr. Sect. A Found. Crystallogr.*, 2008, **64**, 112.

10 C. Vallotto, E. Shaili, H. Shi, J. S. Butler, C. J. Wedge, M. E. Newton and P. J. Sadler, *Chem. Commun.*, 2018, **54**, 13845.

11 N. J. Farrer, J. A. Woods, L. Salassa, Y. Zhao, K. S. Robinson, G. Clarkson, F. S. Mackay and P. J. Sadler, *Angew. Chem. Int. Ed. Engl.*, 2010, **49**, 8905.

12 D. Corinti, M. E. Crestoni, S. Fornarini, F. Ponte, N. Russo, E. Sicilia, E. Gabano and D. Osella, *J. Am. Soc. Mass Spectrom.*, DOI:10.1007/s13361-019-02186-7.

13 M. C. McCormick, K. Keijzer, A. Polavarapu, F. A. Schultz and M. H. Baik, *J. Am. Chem. Soc.*, 2014, **136**, 8992.

14 O. Rivada-Wheelaghan, M. A. Ortuño, S. E. García-Garrido, J. Díez, P. J. Alonso, A. Lledós and S. Conejero, *Chem. Commun.*, 2014, **50**, 1299.
